# A mutation in *DOK7* in congenital myasthenic syndrome forms aggresome in cultured cells, and reduces DOK7 expression and MuSK phosphorylation in patient-derived iPS cells

**DOI:** 10.1093/hmg/ddac306

**Published:** 2022-12-29

**Authors:** Shaochuan Zhang, Bisei Ohkawara, Mikako Ito, Zhizhou Huang, Fei Zhao, Tomohiko Nakata, Tomoya Takeuchi, Hidetoshi Sakurai, Hirofumi Komaki, Masayoshi Kamon, Toshiyuki Araki, Kinji Ohno

**Affiliations:** Division of Neurogenetics, Center for Neurological Diseases and Cancer, Nagoya University Graduate School of Medicine, Nagoya 466-8550, Japan; Division of Neurogenetics, Center for Neurological Diseases and Cancer, Nagoya University Graduate School of Medicine, Nagoya 466-8550, Japan; Division of Neurogenetics, Center for Neurological Diseases and Cancer, Nagoya University Graduate School of Medicine, Nagoya 466-8550, Japan; Division of Neurogenetics, Center for Neurological Diseases and Cancer, Nagoya University Graduate School of Medicine, Nagoya 466-8550, Japan; Division of Neurogenetics, Center for Neurological Diseases and Cancer, Nagoya University Graduate School of Medicine, Nagoya 466-8550, Japan; Division of Neurogenetics, Center for Neurological Diseases and Cancer, Nagoya University Graduate School of Medicine, Nagoya 466-8550, Japan; Division of Neurogenetics, Center for Neurological Diseases and Cancer, Nagoya University Graduate School of Medicine, Nagoya 466-8550, Japan; Center for iPS Cell Research and Application (CiRA), Kyoto University, Kyoto 606-8507, Japan; Department of Pediatrics, National Institute of Neuroscience, National Center of Neurology and Psychiatry, Kodaira, Tokyo 187-8551, Japan; Department of Peripheral Nervous System Research, National Institute of Neuroscience, National Center of Neurology and Psychiatry, Kodaira, Tokyo 187-8551, Japan; Department of Peripheral Nervous System Research, National Institute of Neuroscience, National Center of Neurology and Psychiatry, Kodaira, Tokyo 187-8551, Japan; Division of Neurogenetics, Center for Neurological Diseases and Cancer, Nagoya University Graduate School of Medicine, Nagoya 466-8550, Japan

## Abstract

At the neuromuscular junction, the downstream of tyrosine kinase 7 (DOK7) enhances the phosphorylation of muscle-specific kinase (MuSK) and induces clustering of acetylcholine receptors (AChRs). We identified a patient with congenital myasthenic syndrome (CMS) with two heteroallelic mutations in *DOK7*, c.653-1G>C in intron 5 and c.190G>A predicting p.G64R in the pleckstrin homology domain. iPS cells established from the patient (CMS-iPSCs) showed that c.653-1G>C caused in-frame skipping of exon 6 (120 bp) and frame-shifting activation of a cryptic splice site deleting seven nucleotides in exon 6. p.G64R reduced the expression of DOK7 to 10% of wild-type DOK7, and markedly compromised AChR clustering in transfected C2C12 myotubes. p.G64R-DOK7 made insoluble aggresomes at the juxtanuclear region in transfected C2C12 myoblasts and COS7 cells, which were co-localized with molecules in the autophagosome system. A protease inhibitor MG132 reduced the soluble fraction of p.G64R-DOK7 and enhanced the aggresome formation of p.G64R-DOK7. To match the differentiation levels between patient-derived and control induced pluripotent stem cells (iPSCs), we corrected c.190G>A (p.G64R) by CRISPR/Cas9 to make isogenic iPSCs while retaining c.653-1G>C (CMS-iPSCs^Cas9^). Myogenically differentiated CMS-iPSCs showed juxtanuclear aggregates of DOK7, reduced expression of endogenous DOK7 and reduced phosphorylation of endogenous MuSK. Another mutation, p.T77M, also made aggresome to a less extent compared with p.G64R in transfected COS7 cells. These results suggest that p.G64R-DOK7 makes aggresomes in cultured cells and is likely to compromise MuSK phosphorylation for AChR clustering.

## Introduction

Congenital myasthenic syndromes (CMS) are a heterogeneous group of rare inherited diseases characterized by muscle weakness and fatigue resulting from compromised signal transduction at the neuromuscular junction (NMJ) (1). Mutations in a total of 34 genes have been reported to cause CMS (2,3). Molecules in the agrin-LRP4-MuSK-DOK7 signaling pathway are essential for the clustering of acetylcholine receptors (AChRs) at the motor endplate (4–10). Both defective and excessive phosphorylation of muscle-specific kinase (MuSK) reduces AChR clusters (11). The downstream of tyrosine kinase 7 (DOK7) enhances agrin-triggered MuSK phosphorylation (8,9). In the absence of *Dok7* in mice, agrin cannot induce AChR clustering (12). Phosphorylated MuSK also recruits DOK7 and induces DOK7 phosphorylation. This positive feedback loop ensures sufficient activation of MuSK and triggers AChR clustering. DOK7 is comprised of a pleckstrin homology (PH) domain, a phosphotyrosine-binding (PTB) domain and a long unstructured C-terminal region containing a nuclear exporting signal (NES) and two tyrosine residues that can be phosphorylated. The PH domain enables the anchoring of DOK7 to the plasma membrane and the assembly of a protein complex with MuSK (13). The PTB domain contains a MuSK-binding motif (9). The integrity of the PH-PTB domains is critical for dimerizing DOK7 to facilitate MuSK phosphorylation (14). The NES domain controls the subcellular localization of DOK7. Two tyrosine residues provide binding sites for the adaptor proteins such as Crk and Crk-L (15,16), which mediate the downstream signaling pathway to induce AChR clustering at the NMJ.

*DOK7* mutations account for 10–15% of CMS, and are the most common cause of limb-girdle myasthenia (8,15,17). More than 70 missense, truncation and splicing mutations have been reported in *DOK7* in CMS (8,15,17,18). Mutations in *DOK7* cause aberrantly small and simplified neuromuscular synapses (8). Patients with *DOK7* mutations usually respond to ephedrine or salbutamol, but often worsen with cholinesterase inhibitors that are effective in most types of CMS (19,20). Twelve missense mutations in *DOK7* (p.E3K, p.P31T, p.A33V, p.S45L, p.T77M, p.G109C, p.V139L, p.R158Q, p.G161R, p.G166R, p.G171D and p.G180A) have been characterized in overexpression systems in two reports (15,21). They variably reduced the phosphorylations of MuSK and AChR ß1 subunit, but none reduced the expression of DOK7. Formation of aggregates by mutant DOK7 has not been investigated to date.

**Figure 1:**
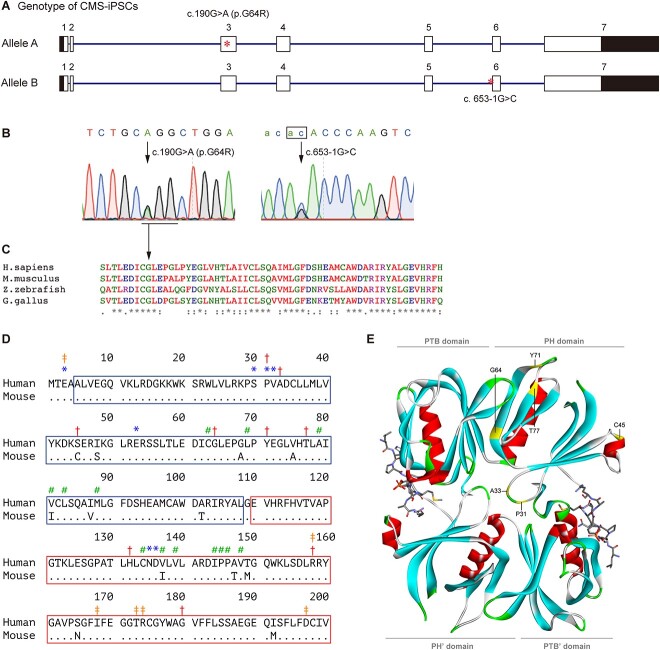
Mutations in *DOK7*. (**A**) Schematic of *DOK7* gene (NM_173660.5) showing the location of mutations (red asterisks). The 5′ and 3′ untranslated regions are shown in black. (**B**) Sequencing chromatograms showing heteroallelic mutations. An invariant ‘ag’ dinucleotide at the 3′ end of intron 5 is indicated by a box. (**C**) Alignment of DOK7 proteins across species. Alignment was performed using ClustalW2 (https://www.ebi.ac.uk/Tools/msa/clustalw2/). Glycine at codon 64 (GGG) is conserved across species, and is changed to arginine (AGG). (**D**) Alignment of the PH (blue box) and PTB (red box) domains of human and mouse DOK7 (14). Identical residues are indicated by dots. ^*^Residues for DOK7 dimerization. #Residues for PH-PTB interaction. ‡Residues for binding to MuSK pTyr533. †Residues mutated in CMS. Note that the mutated serine at codon 45 in human is cysteine at codon 45 in mouse. (**E**) Crystal structure of the PH and PTB domains of mouse DOK7 dimer (ribbons) and MuSK phosphopeptides (sticks) (14). Six residues mutated in CMS in the PH domain are indicated in the upper DOK7. Domains in the lower DOK7 are indicated by apostrophe.

Here, we characterized two mutations, c.653-1G > C and c.190G > A, in *DOK7* in a patient with CMS (Fig. 1A and B). c.653-1G > C generated two aberrantly spliced transcripts in CMS-induced pluripotent stem cells (iPSCs). In transfected COS7 cells and C2C12 myoblasts/myotubes, p.G64R reduced the expression of DOK7 by forming insoluble aggregates, and compromised MuSK phosphorylation and AChR clustering. In CMS-iPSCs, p.G64R formed juxtanuclear aggregates to a less extent and reduced the expression of DOK7 and MuSK phosphorylation, which were rescued by correcting p.G64R by CRISPR/Cas9.

## Results

### CMS patient

A male patient, currently at age 24 years, had respiratory distress and muscle weakness at birth. He had undergone tracheotomy and was dependent on a respirator up to age 4 years. He had ptosis and limitations in eye movements, which disappeared over time, but the exact temporal profile was not recorded. At age 4 years, he started walking independently, but he showed marked day-to-day variability from being unable to walk to being able to climb 10 steps of stairs. He showed no diurnal fluctuation of muscle weakness. Currently, proximal dominant muscle weakness is observed, but the patient is able to walk independently. Repetitive nerve stimulation decreased the amplitude of compound muscle action potential to 79% of the accessory nerve, but not of the median nerve. Biopsy of biceps brachii at age 9 years showed a decrease of type IIB fibers and an increase of type IIC fibers, but no tubular aggregates. Ephedrine hydrochloride and 3, 4-diaminopyridine showed moderate effects.

### Mutations in *DOK7*

Sanger sequencing revealed that the patient was heterozygous for two mutations in *DOK7*. One was c.190G > A (NM_173660.5) at position 3 473 495 (GRCh38/hg38) on chromosome 4, predicting p.G64R (NP_775931.3) in the PH domain (Fig. 1A and B). p.G64 is highly conserved across species (Fig. 1C). p.G64R was predicted to be highly pathogenic by InMeRF with a probability of 0.890 (22) (Supplementary Material, Table S1). p.G64R has an accession number of rs1246160310 in dbSNP with global minor allelic frequency (GMAF) = 0.0011% (3/266842). p.G64R was previously reported in two unrelated CMS patients without functional characterization (23). p.G64 was located at the interface between the PH and PTB domains in dimerized DOK7 (Fig. 1D and E) (14).

Another mutation was c.653-1G > C (NM_173660.5) at position 3 491 403 (GRCh38/hg38) on chromosome 4, at the 3′ end of intron 5 (Fig. 1A andB). c.653-1G > C has an accession number of rs1449351306 with GMAF = 0.006% (1/16760). c.653-1G > C was reported in another *DOK7*-CMS patient without functional characterization (24).

Sanger sequencing showed that the asymptomatic father and mother were heterozygous for c.653-1G > C and c.190G > A, respectively.

### Activation of a cryptic 3′ splice site in *DOK7* exon 6 due to c.653-1G > C at the 3′ end of intron 5

**Figure 2:**
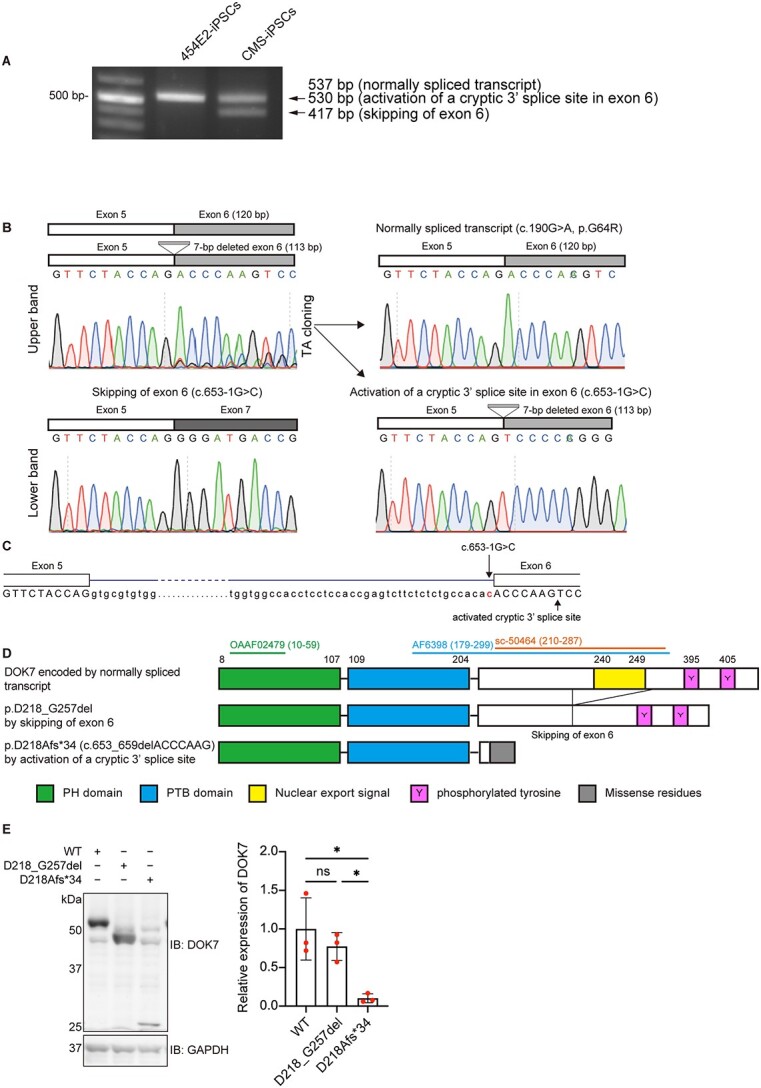
Aberrantly spliced transcripts due to c.653-1G > C. (**A**) RT-PCR spanning *DOK7* exon 6 of control 454E2-iPSCs and patient-derived CMS-iPSCs. The upper band was comprised of 537-bp and 530-bp fragments. (**B**) Sequence chromatograms of three RT-PCR products in CMS-iPSCs. Chromatograms of the two transcripts in the upper band in A are indicated on the right. (**C**) Schematic showing the positions of the c.653–1 > C mutation and activated cryptic 3′ splice site that is seven nucleotides downstream of the intron 5/exon 6 junction. (**D**) Schematic presentation of DOK7 proteins encoded by three transcripts in B. Epitopes of three DOK7 antibodies (OAAF02479, AF6398 and sc-50464) used in this study are indicated with codon numbers in parentheses. See Supplementary Material, Table S2 for the details of these antibodies and Supplementary Material, Fig. S5A for representative western blotting. (**E**) Representative western blotting and quantitative analysis of mutant DOK7 arising from an allele with c.653-1G > C in transfected COS7 cells. Expression levels were normalized to that of GAPDH and to the ratio of wild-type (WT) DOK7. Mean and SD (*n* = 3 experiments) are indicated with individual values in red dots. One-way ANOVA with Dunnett’s post hoc multiple comparison test was applied (ns, no significance; ^*^P < 0.05).

To examine the effects of c.653-1G > C on splicing, we analyzed *DOK7* transcripts in CMS-iPSCs (Fig. 1). Reverse transcription-polymerase chain reaction (RT-PCR) spanning *DOK7* exon 6 (120 bp) and sequencing of cloned fragments showed two aberrantly spliced transcripts in CMS-iPSCs (Fig. 2A). One was due to skipping of exon 6, and the other was due to an activation of a cryptic splice site deleting seven nucleotides at the 5′ end of exon 6 (Fig. 2B andC). The exon 6-skipped transcript was more abundant than the 7-nt-deleted transcript.

The exon 6-skipped *DOK7* transcript has an in-frame deletion lacking codons 218–257 (p.D218_G257del), where the nuclear export signal (NES) at 240–249 is located (Fig. 2D). Although p.D218_G257del-DOK7 was similarly expressed compared with WT-DOK7 in COS7 cells (Fig. 2E), disruption of the NES domain was previously reported to compromise MuSK phosphorylation and impair AChR clustering in C2C12 myotubes (15,25). The 7-nt-deleted transcript (c.653_659delACCCAAG) predicts p.D218Afs^*^34 (Fig. 2D), the expression level of which was markedly reduced in COS7 cells (Fig. 2E). These results suggested that c.653-1G > C was likely to generate DOK7 with markedly compromised functions.

### Effects of p.G64R on protein expression and AChR clustering

**Figure 3:**
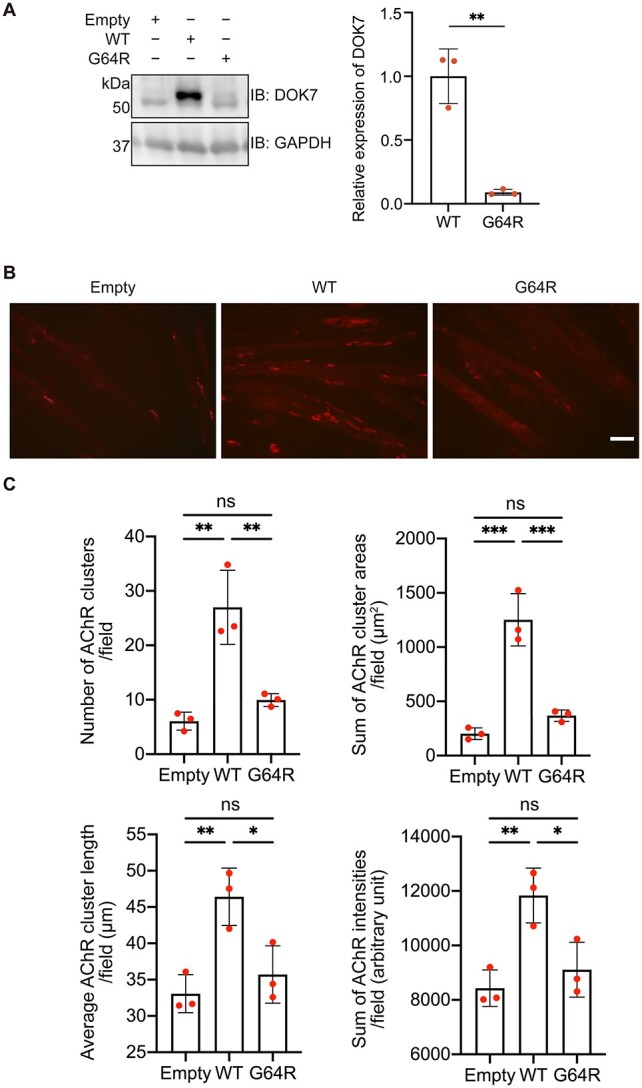
Effects of p.G64R-DOK7 on protein expression and AChR clustering in C2C12 cells. (**A**) Representative western blotting and quantitative analysis of DOK7 in C2C12 cells transfected with wild-type (WT)-DOK7 or p.G64R-DOK7. Mean and SD (*n* = 3 experiments) are indicated with individual values in red dots. Student’s *t*-test (^*^^*^P < 0.01). (**B**) Transfection of wild-type (WT)-DOK7 but not p.G64R-DOK7 induced AChR clustering visualized by Alexa 594-conjugated α-bungarotoxin (red) in C2C12 cells without agrin. Scale bar = 50 μm. (**C**) Quantitative analysis of the number, total area, average length and total signal intensity per visual field (0.143 mm^2^) of AChR clusters in C2C12 cells expressing WT-DOK7 or p.G64R-DOK7. p.G64R-DOK7 markedly reduced AChR clustering. The mean values of 30 visual images are indicated by red dots. One-way ANOVA and Dunnett’s multiple comparison test (ns, no significance; ^*^P < 0.05, ^*^^*^P < 0.01 and ^*^^*^^*^P < 0.001).

First, we analyzed the expression level of p.G64R-DOK7. Western blotting of transfected C2C12 myoblasts showed that p.G64R significantly reduced the expression of DOK7 (Fig. 3A). Second, we examined the binding of DOK7 to MuSK and the phosphorylation of MuSK. As C2C12 myotubes express DOK7 endogenously, we used COS7 cells that express no or negligible amounts of DOK7 and MuSK (15). p.G64R had no effect on the protein level of MuSK, but reduced the amount of MuSK co-immunoprecipitated with DOK7 in COS7 cells that were transfected with FLAG-tagged MuSK along with WT-DOK7 or p.G64R-DOK7 (Supplementary Material, Fig. S1A). Similarly, tyrosine phosphorylation of MuSK was markedly reduced in p.G64R-DOK7-transfected COS7 cells (Supplementary Material, Fig. S1A). Third, we analyzed the effects of p.G64R on AChR clustering using C2C12 myotubes. WT-DOK7 induced AChR clustering in the absence of agrin, but p.G64R-DOK7 had no essential AChR clustering activity in transfected C2C12 myotubes (Fig. 3B andC). These results suggested that p.G64R reduced the expression of DOK7, and compromised the DOK7-mediated MuSK phosphorylation and AChR clustering.

### Ubiquitin inhibitor MG132 accelerates the reduction of soluble p.G64R-DOK7 through forming insoluble aggregates

**Figure 4:**
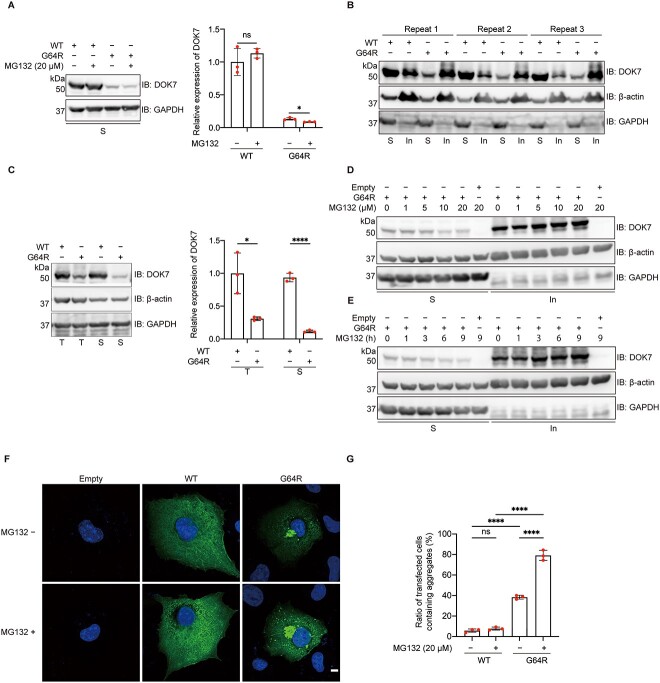
Effects of p.G64R-DOK7 on protein solubility. (**A** and **C**) Representative western blotting and quantitative analysis of DOK7 and GAPDH without ß-actin (A) or with ß-actin (C) in detergent-soluble fractions (S) and total protein lysates (T), in transfected COS7 cells. MG132 (20 μM) was added 3 h before harvesting cells (A). Expression levels were normalized to that of GAPDH, and also to the ratio of wild-type (WT)-DOK7. Mean and SD (*n* = 3 experiments) are indicated with individual values in red dots. Multiple Student *t*-test was applied (ns, no significance, ^*^P < 0.05, ^*^^*^^*^^*^P < 0.0001). Triplicated (**B**) and representative (**D** and **E**) western blotting of DOK7, ß-actin and GAPDH in detergent–soluble (S) and insoluble (In) fractions, in transfected COS7 cells. (D) MG132 at 0–20 μM was added 3 h before harvesting cells. (E) MG132 at 20 μM was added at indicated time points. (**F**) Representative images of WT-DOK7 and p.G64R-DOK7 treated with or without 20 μM MG132 treatment for 3 h in transfected COS7 cells. Green and blue signals represent DOK7 and DAPI, respectively. Scale bar = 10 μm. (**G**) The ratio of transfected COS7 cells with aggregates in each experiment is indicated in red dot. Each experiment is an average of five visual fields and is comprised of at least 100 cells. Mean and SD (*n* = 3 experiments) are indicated. One-way ANOVA with Dunnett’s multiple comparison test (ns, no significance; ^*^^*^^*^^*^P < 0.0001).

We next examined whether the reduced expression of p.G64R-DOK7 was due to (i) the reduced amount of mRNA, (ii) the accelerated degradation of DOK7 [e.g. degradation by the ubiquitination proteasome system (UPS)] or (iii) the formation of detergent-insoluble aggregates. First, quantitative RT-PCR of *DOK7* transfected into COS7 cells showed that WT- and p.G64R-DOK7 were similarly transcribed, and the addition of a proteasome inhibitor, MG132, had no effect on mRNA levels (Supplementary Material, Fig. S1B). Second, treatment of transfected COS7 cells with MG132 for 3 h decreased the amount of p.G64R-DOK7 (Fig. 4A) in a dose-dependent manner with an apparent half-life of 3.3 h (Supplementary Material, Fig. S1C and D). This indicated that an accelerated UPS-mediated degradation of DOK7 was not the cause of the reduced expression of p.G64R-DOK7. Third, we examined whether p.G64R form a detergent-insoluble fraction. WT-DOK7 was mostly present in the detergent-soluble fraction, whereas p.G64R-DOK7 was mostly located in the detergent-insoluble fraction (Fig. 4B). p.G64R also decreased the sum of the soluble and insoluble fractions of DOK7 (Fig. 4C). MG132 treatment increased the amount of p.G64R-DOK7 in the insoluble fraction in dose- and time-dependent manners (Fig. 4D andE). Immunostaining of DOK7 in COS7 cells expressing WT- and p.G64R-DOK7 also showed that p.G64R-DOK7 was prone to form aggregated puncta at the juxtanuclear region, whereas WT-DOK7 was diffusely dispersed in the cytoplasm (Fig. 4F andG). Thus, p.G64R-DOK7 that could not be degraded by the UPS was sequestrated into the insoluble fraction, which subsequently reduced the expression of p.G64R-DOK7.

In addition to COS7 cells, we also evaluated the aggregates of p.G64R-DOK7 in transfected C2C12 myoblasts. We observed that enhanced green fluorescent protein (EGFP)-fused p.G64R-DOK7 formed aggregates at the juxtanuclear region in C2C12 myoblasts (Supplementary Material, Fig. S2). Addition of the PLC lysis buffer to transfected C2C12 myoblasts dispersed the cytoplasmic green signals of WT-DOK7-EGFP, but not of aggregates made of p.G64R-DOK7-EGFP, indicating that the aggregates were detergent-insoluble (Supplementary Material, Video).

These results suggested that the reduced expression of p.G64R-DOK7 was accounted for by the formation of abnormal aggregates at the juxtanuclear region.

### Characterization of aggregates formed by p.G64R-DOK7

**Figure 5:**
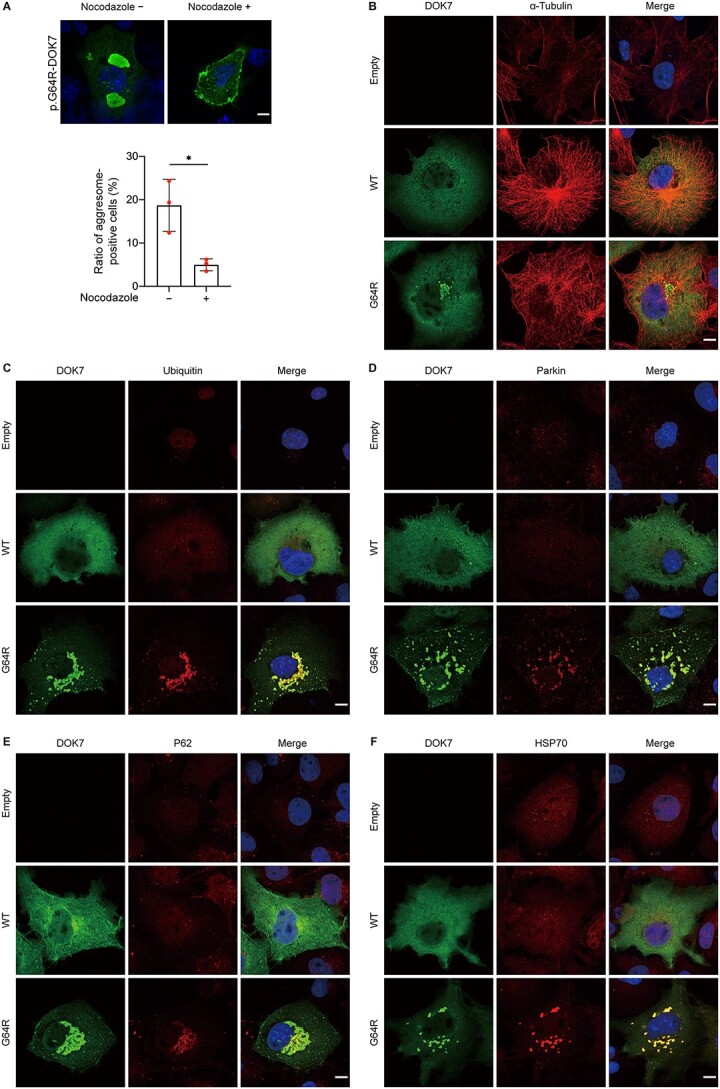
Characterization of aggregates of p.G64R-DOK7. (**A**) Representative immunostaining of p.G64R-DOK7 in transfected COS7 cells with or without 10 μM nocodazole, an inhibitor of microtubule polymerization. Scale bar = 10 μm. The ratio of aggresome-positive cells after 10 μM nocodazole treatment for 3 h in five visual fields is plotted in red for each experiment. Mean and SD (*n* = 3 experiments) are indicated. ^*^P < 0.05 by Student’s *t*-test. (**B-E**) Co-localization of DOK7 and aggresome markers of α-tubulin (B), ubiquitin (C), parkin (D), P62 (E) and HPS70 (F). COS7 cells transfected with wild-type (WT)-DOK7 or p.G64R-DOK7 were immunostained with indicated antibodies at 18 h after transfection. Scale bar = 10 μm.

The UPS is primarily responsible for eliminating unfolded and misfolded proteins. When the load of UPS is overwhelmed, protein aggresomes are formed at the microtubule-organizing center (MTOC) in a microtubule-dependence manner (26). We thus examined the association of the aggregates with molecules in the MTOC. Nocodazole, an inhibitor of microtubule formation, markedly reduced the formation of aggresomes of p.G64R-DOK7 in transfected COS7 cells (Fig. 5A). A microtubule component, α-tubulin (Fig. 5B) as well as the other aggresome markers (ubiquitin, parkin, P62, and HSP70) (Fig. 5C–F) were colocalized with p.G64R-DOK7 aggregates. These results suggested that p.G64R caused abnormal folding of DOK7, which made DOK7 ubiquitinated, transported to MTOC and form aggresomes at the juxtanuclear region.

### Comparison of CMS-iPSCs with p.G64R and isogenic CMS-iPSCs ^Cas9^ without p.G64R

**Figure 6:**
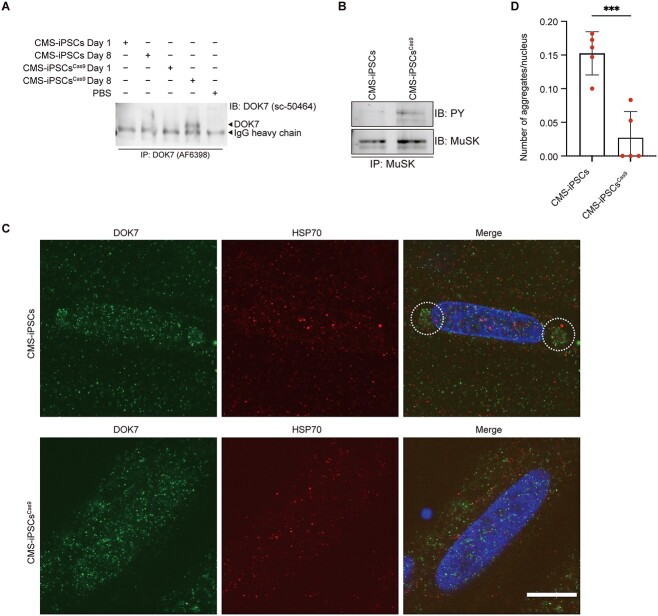
The expression level of DOK7, the phosphorylation of MuSK and the formation of aggregates in patient-derived CMS-iPSCs and isogenic CMS-iPSC^Cas9^**.** (**A**) Representative western blotting of DOK7 in myogenically differentiated iPSCs on Day 8. Low expression level of DOK7 necessitated the enrichment of DOK7 by immunoprecipitation (IP) before immunoblotting (IB). Anti-DOK7 antibodies are indicated in parentheses. (**B**) Phosphorylated MuSK was immunoblotted (IB) by an anti-phosphotyrosine antibody (PY) after immunoprecipitation (IP) of MuSK. (**C**) Representative immunostaining of DOK7 and HSP70 in CMS-iPSC- and CMS-iPSC^Cas9^-derived myogenic cells. Juxtanuclear aggregates are indicated by dotted circles. Scale bar = 10 μm. (**D**) The number of juxtanuclear aggregates was blindly counted in five visual fields (~30 nuclei each). Mean and SD are plotted. ^^*^^*^^*^^P = 0.005 by Student’s *t*-test.

To dissect the effect of p.G64R in patient-derived iPSCs, we established an isogenic cell line, CMS-iPSCs^Cas9^, from CMS-iPSCs by correcting c.190A > G (p.G64R), while retaining c.653-1G > C on another allele (Supplementary Material, Fig. S3A–C). First, myogenic marker genes (*MYH3* and *MYOD1*) and NMJ-associated genes (*LRP4*, *DOK7*, *MUSK* and *CHRNG*) were induced in myogenic differentiation of both CMS-iPSCs and CMS-iPSCs^Cas9^ (Supplementary Material, Fig. S3D). Next, we observed that, in contrast to CMS-iPSCs^Cas9^, endogenous DOK7 was undetectable even after immunoprecipitation (Fig. 6A), and the phosphorylation of endogenous MuSK was markedly low in myogenically differentiated CMS-iPSCs (Fig. 6B andSupplementary Material, Fig. S4A). Although the staining of endogenous DOK7 was markedly weak in myogenically differentiated CMS-iPSCs (Supplementary Material, Fig. S4B), DOK7 made aggregates at the juxtanuclear region (Fig. 6C andD). In contrast to COS7 cells, however, HSP70 was not colocalized with DOK7. Taken together, p.G64R reduced the expression of DOK7 and MuSK phosphorylation and formed aggregates at the juxtanuclear region in myogenically differentiated CMS-iPSCs.

Unavailability of two anti-DOK7 antibodies, where one was for immunoprecipitation and the other was for immunoblotting, prevented us from evaluating abnormal DOK7 originating from an allele with c.653-1G > C in CMS-iPSCs (Supplementary Material, Fig. S5 and Supplementary Material, Table S2).

### Prediction and observation of aggresome formations by five missense mutations at the PH domain

**Figure 7:**
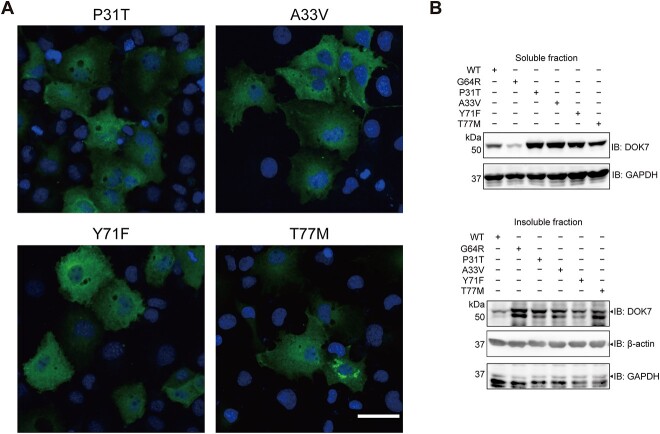
Immunostaining and western blotting of four other missense mutants at the PH domain of DOK7 in COS7 cells. (**A**) Representative immunostaining of DOK7 in COS7 cells transfected with indicated mutants. Refer to Figure 4I for representative images of wild-type and p.G64R-DOK7. Scale bar = 50 μm. (**B**) Western blotting of DOK7 in soluble (upper) and insoluble (lower) fractions in transfected COS7 cells.

As shown in Figure 1D and E, six missense mutations (p.P31T, p.A33V, p.S45L, p.G64R, p.Y71F and p.T77M) have been reported in CMS at the PH domain. Except for p.S45L that was not conserved between mouse and human (Fig. 1D), the effects of five missense mutations on protein stability and protein structure were evaluated by eight tools (Supplementary Material, Table S3a). Among the five missense mutations, ΔΔG of p.G64R, which is a hallmark of the effect on the protein instability (27,28), was ranked first or second in most tools (Supplementary Material, Table S3b). Generation of novel aggregation-prone regions (APRs) by p.G64R was also predicted (Supplementary Material, Fig. S6A and B). In addition, substantial structural changes of p.G64R, as well as p.T77M to a less extent, were predicted (Supplementary Material, Table S3c). Furthermore, p.G64R was predicted to form new interactions with other amino acids (Supplementary Material, Fig. S6C). We indeed observed that, among the five missense mutations, p.T77M also formed juxtanuclear aggresome to a less extent compared with p.G64R (Fig. 7), but the expression level of p.T77M was not reduced. Thus, the formation of aggresome is not unique to p.G64R-DOK7, and p.T77M can also disrupt DOK7 folding and form aggresome.

## Discussion

We identified compound heterozygous mutations (c.653-1G > C and p.G64R) in *DOK7* in a patient with CMS. *DOK7*-CMS is classified into limb-girdle myasthenia, but a review of 15 patients with *DOK7*-CMS showed the presence of ocular involvement in 11 patients (73%) (29), as we observed in our patient. The deletion of the C-terminal region of DOK7 in one or two alleles is observed in most *DOK7*-CMS patients, which markedly reduces the expression levels of DOK7 (18). In addition, the deletion of the C-terminal region and the disruption of the NES domain in DOK7 compromise DOK7-mediated MuSK phosphorylation and impair AChR clustering in C2C12 myotubes (15,25). We also observed that the C-terminal truncated DOK7 by c.653-1G > C markedly reduced the protein expression level. In contrast to *Dok7*-deficient mice that showed no AChRs in the diaphragm (2,3), mice carrying the PH and PTB domains by knocking in homozygous c.1124_1127dupTGCC can form immature AChRs with small size, less number and simplified structure (30). Similarly, *DOK7*-CMS patients with homozygous c.1124_1127dupTGCC show severe symptoms but are not lethal (30). Therefore, lack of the C-terminal region of DOK7 largely compromises the NMJ formation, but does not nullify the effect of DOK7.

We observed that un- or mis-folded p.G64R-DOK7 caused an overload to the UPS, and unprocessed p.G64R-DOK7 made aggresomes at the MTOC. Similar activation of the autophagosome system by an overload the UPS has been repeatedly reported and is reviewed (26). Myogenically differentiated CMS-iPSCs showed that DOK7 made aggregates at the juxtanuclear region without colocalization of HPS70 and that DOK7 expression and MuSK phosphorylation were markedly reduced. CRSPR/Cas9-medited correction of p.G64R in CMS-iPSCs rescued these phenotypes. Lack of aggresomes in CMS-iPSCs was likely to be accounted for by high UPS activities in iPSCs (31–33). Alternatively, this was due to a technical constraint that iPSCs could not form mature myotubes compared with C2C12 myoblasts (34). Although aggresomes were observed in cultured cells and possibly in CMS-iPSCs, no aggregates were observed by hematoxylin-eosin (HE) staining of biopsied skeletal muscle at Age 9, and the disease did not progress in 15 years after the biopsy, the roles of aggresomes in the patient remain elusive.

Small molecule chaperones, cystic fibrossis transmembrane conductance regulator (CFTR) correctors, have been successfully applied to and approved for reverting folding defects of the CFTR-ΔF508 mutant in cystic fibrosis (35–37). CFTR correctors are unique in that most small molecule chaperones were developed as competitive inhibitors, whereas CFTR correctors enhance protein folding (38). Similar small molecule chaperons may be able to correct misfolding of mutant DOK7 due to p.G64R and others.

## Materials and Methods

### Generation of CMS-iPSCs

CMS-iPSCs were generated from the peripheral blood monocytes of a patient. All clones were generated using episomal vectors as previously described (39,40). Control iPSCs (454E2-iPSCs) were obtained from the Center for iPS Cell Research and Application, Kyoto University. The established CMS-iPSCs and 454E2-iPSCs were cultured in Primate ES Cell medium (RCHEMD001, Repro CELL) supplemented with 8 ng/ml basic fibroblast growth factor (RCHEOT003, Repro CELL) and 1× Zell Shield (13–0050, Minerva Biolabs) on mitomycin C-treated SNL feeder cells, as described previously (41). For establishment of feeder-free culture of iPSCs, feeder-dependent cultures were adapted into Essential 8 Flex medium (E8-Flex, A28585, Thermo Fisher Scientific) according to the manufacturer’s protocol. For passaging, the cells were treated with 1 mg/ml Collagenase IV solution (C4–28, Sigma-Aldrich) and gravity-sedimented. The cells were then suspended in E8-Flex and distributed on vitronectin-(VTN-N, A14700, Thermo Fisher Scientific) coated dishes containing E8-Flex medium with 10 μM Y-27634. For differentiation into skeletal muscle cells, doxycycline (Dox)-inducible MyoD expression vector and PBase vector were electroporated into CMS-iPSCs and 454E2-iPSCs using a NEPA21 type II electroporator (Nepa Gene) (42). The transduced cells were selected with puromycin (P8833, Sigma-Aldrich) (1 μg/ml for CMS-iPSCs or 125 ng/ml for 454E2-iPSCs) and clones showing uniformly high MYOD1 expression were selected. The gene expressions of pluripotency markers, *POU5F1* (Oct3/4), *SOX2*, *NANOG* and *MYC* (c-myc) were detectable in undifferentiated CMS-iPSCs and 454E2-iPSCs by RT-PCR (Supplementary Material, Fig. S8A). After 8 days of myogenic differentiation, myotube-like spindle-shaped cells with multiple nuclei were observed (Supplementary Material, Fig. S8B and C). There was no significant difference in the morphology in both clones.

### Generation of isogenic control CMS-iPSCs^Cas9^

Genomic sequences of *DOK7* exons 3 and 6 in CMS-iPSCs were first determined by Sanger sequencing to confirm the presence of heterozygous c.653-1G > C and c.190G > A mutations. To make isogenic control iPSCs, c.190G > A (p.G64R) in *DOK7* exon 3 in CMS-iPSCs was corrected by CRISPR/Cas9 in an allele-specific manner (Supplementary Material, Fig. S9), while retaining c.653-1G > C on another allele. The guide RNA (gRNA) was designed against allele A carrying c.190G > A using CHOPCHOP (https://chopchop.cbu.uib.no/). The c.190G > A mutation was located 4 bp upstream of the protospacer adjacent motif (PAM) site. The sequence of an 81-nt single-strand oligodeoxynucleotide (ssODN) was: 5′-CCAGCGTGTGGACCAGGCCCTCGTAGGGCAGGCCGGGCTCgAGCCcGCAGATGTCCTCTAGCGTCAGGCTGCTGCGCTCCC-3′, where the complementary sequence shown in Supplementary Material, Fig. S8A is underlined. The ssODN carried c.190G to correct the mutation (lowercase letter ‘c’), as well as an artificial silent c.195G > C mutation (lowercase letter ‘g’) at the PAM site, which also introduced an Xhol restriction site. Cas9 protein (Thermo Fisher Scientific), gRNA and ssODN were introduced into iPSCs by electroporation following a previously reported protocol (43). Electroporated cells were cultured in two dishes. At 48 h after transfection, DNA was extracted from a single dish, and a PCR fragment spanning c.190G > A was digested by *Xho*I to confirm the presence of cells carrying the artificial silent mutation. Next, 200–400 cells were plated on a 10-cm dish for isolation of single colonies for 7–10 days. Single colonies were picked up, and further expanded in 48-well plates. Mutation-corrected cells (CMS-iPSCs^Cas9^) were identified by an allele-specific PCR (ASP) primer, 5′-GCCTGACGCTAGAGGACATCTGCgGGgTc-3′, where ‘g’ at position −6 was for recognizing the corrected c.190G, ‘g’ at position −3 was an artificial mismatch against the target cDNA for better discrimination in ASP and ‘c’ at the 3′ end was at the artificial silent mutation at the PAM site (Supplementary Material, Table S3). For ASP-positive colonies, the top five off-target sites, which were predicted by CHOPCHOP (https://chopchop.cbu.uib.no/), were Sanger-sequenced, and no artificial mutation was observed. We confirmed that CMS-iPSCs^Cas9^ had the expected correction (Supplementary Material, Fig. S3A), and expressed all pluripotency markers as in CMS-iPSCs (Supplementary Material, Fig. S3B). There were no significant differences in morphology during the myogenic differentiation (Supplementary Material, Fig. S3C).

### Culture of CMS-iPSCs

iPSCs were cultured on a plate coated with Vitronectin (VTN-N, A14700, Thermo Fisher Scientific) in Essential-8 Medium (E8, A2858501, Thermo Fisher Scientific) containing puromycin (1 μg/ml for CMS-iPSCs or 125 ng/ml for 454E2-iPSCs) and 10 μM Y-27632 (030–24 021, Fujifilm Wako Chemicals). Cells were seeded at a density of 14 000 cells per well in a 6-well plate and passaged every 5–7 days using Accutase (A1110501, Thermo Fisher Scientific). Medium was changed every day.

For myogenic differentiation, the cells were collected with Accutase, and seeded on a plate coated with Matrigel (Corning) in E8 with puromycin and Y-27632 (Supplementary Material, Fig. S7). On the next day (differentiation Day 1), the medium was replaced with Dulbecco’s-modified Eagle’s medium (DMEM)/F12 (11 320 033, Thermo Fisher Scientific) supplemented with 5% KnockOut Serum Replacement (KSR, 10828028, Thermo Fisher Scientific), 10 μM SB431542 (SB, 13031, Cayman Chemical), 2 μM dorsomorphin (Dor, 3093, R&D Systems), 0.5 mM N-acetyl-L-cysteine (NAC, A7250, Sigma-Aldrich) and 3 μM CHIR99021 (CH, R&D Systems). At 24 additional hours (differentiation Day 2), the medium was changed to DMEM/F12 containing 5% KSR, SB, Dor, NAC and 1 μM retinoic acid (RA, R2625, Sigma-Aldrich). On differentiation Day 3, the medium was changed to DMEM/F12 with 5% KSR and 1 μg/ml Dox. On differentiation Day 4, the medium was changed to Minimum essental medium α (αMEM) (2 144 405, Nacalai Tesque) with 5% KSR, 100 μM 2-mercaptoethanol (2-ME) and 1 μg/ml Dox. On differentiation Day 5, the medium was changed to the fresh αMEM. On differentiation Day 6, cells were dissociated with Accutase and seeded on Matrigel-coated plates at a density of 1.0 × 10^6^ cells per well in a 6-well plate in αMEM, 5% KSR, 100 μM 2-ME and 1 μg/ml Dox. On differentiation Day 7, cells were added with fresh medium with the same ingredients and were cultured for additional 24 h.

### Transcriptomic and immunostaining analysis of CMS-iPSCs

Splicing pattern due to the c.653-1G > A mutation was determined by Sanger sequencing of transcripts spanning *DOK7* exon 6. Primer sequences are shown in Supplementary Material, Table S4. For immunofluorescent staining of DOK7 in CMS-iPSCs, 1.0 × 10^6^ cells were plated on Matrigel-coated glass coverslips in each well of 6-well plate.

### Analysis of AChR clustering in C2C12 myotubes transfected with wild-type and mutant *DOK7*

C2C12 myoblasts were cultured in DMEM supplemented with 10% fetal bovine serum (Thermo Fisher Scientific) at 37°C with 5% CO_2_. One day before transfection, 1.0 × 10^5^ cells were plated in each well of a collagen I-coated 6-well plate. On the following day, a plasmid encoding WT-DOK7 or p.G64R-DOK7 was transfected into C2C12 cells using lipofectamine 3000 (Thermo Fisher Scientific) according to the manufacturer’s instructions. After 24 h, the medium was switched to DMEM with 2% horse serum (Thermo Fisher Scientific) to induce myogenic differentiation. After 5–7 days of differentiation, agrin (10 ng/ml) was added to induce AchR clustering for 18 h. To observe AchR clusters, cells were incubated with Alexa Flour 594-tagged α-bungarotoxin for 1 h. Then, cells were fixed for 10 min using phosphate-buffered saline (PBS) containing 4% formaldehyde. Images were captured under an Olympus XL71 fluorescence microscope. The number, size and signal intensity of AchR clusters were analyzed using Image J software (http://imagej.nih.gov/ij/) with an automated macro (Supplementary Material, Fig. S10).

### Analysis of detergent-soluble and insoluble fractions

Detergent-soluble and insoluble fractions were extracted as described previously with minor modifications (44). Briefly, cells were lysed in 60 μL PLC buffer supplemented with protease inhibitors. After rotation at 4°C for 30 min, the lysates were centrifuged for 5 min at 15 000 × *g* at 4°C. The supernatants were designated as detergent-soluble proteins and transferred to new tubes. The pellets containing detergent-insoluble proteins were washed with PLC buffer and dissolved in 60 μL urea buffer (2% SDS, 6 M urea and 30 mM Tris–HCl pH 7.6), followed by sonication on ice for 10 s. Equal volumes of protein extraction were heated at 95°C for 5 min in 2× Laemmli buffer and analyzed by western blotting.

### Statistical analysis

Values are represented by mean and SD. Data were analyzed by Student’s *t*-test, multiple Student’s *t*-test or one-way ANOVA followed by Dunnett’s posthoc test. *P*-values < 0.05 were considered to be statistically significant.

## Genomic sequencing of the patient

Details are indicated in Supplementary Materials and Methods.

## Expression analysis of wild-type and mutant DOK7 in COS7 cells

Details are indicated in Supplementary Materials and Methods.

## Coimmunoprecipitation

Details are indicated in Supplementary Materials and Methods.

## Plasmid constructions

Details are indicated in Supplementary Materials and Methods.

## mRNA expression analysis by reverse-transcription PCR (RT-PCR) and real-time RT-PCR

Details are indicated in Supplementary Materials and Methods.

## Western blotting

Details are indicated in Supplementary Materials and Methods.

## Immunofluorescence staining and confocal microscope

Details are indicated in Supplementary Materials and Methods.

## Supplementary Material

Sup_Figure_1_ddac306

Sup_Figure_2_ddac306

Sup_Figure_3_ddac306

Sup_Figure_4_ddac306

Sup_Figure_5_ddac306

Sup_Figure_6_ddac306

Sup_Figure_7_ddac306

Sup_Figure_8_ddac306

Sup_Figure_9_ddac306

Supplementary_Table_S1_ddac306

Supplementary_Table_S2_ddac306

Supplementary_Table_S3_ddac306

Supplementary_Table_S4_ddac306

Legends_for_Supplementary_Figures_ddac306

Supplementary_Materials_and_Methods_ddac306

C2C12_transfected_with_GFP-DOK7_WT_and_add_PLC_ddac306

C2C12_transfected_with_GFP-DOK7_G64R_and_add_PLC_ddac306
