# Supplementary figures and images for: A mutation in DOK7 in congenital myasthenic syndrome forms aggresome in cultured cells, and reduces DOK7 expression and MuSK phosphorylation in patient-derived iPS cells
Source: Hum Mol Genet. 2022 Dec 29;32(9):1511–23. doi: 10.1093/hmg/ddac306 (PMC10117378; doi:10.1093/hmg/ddac306)

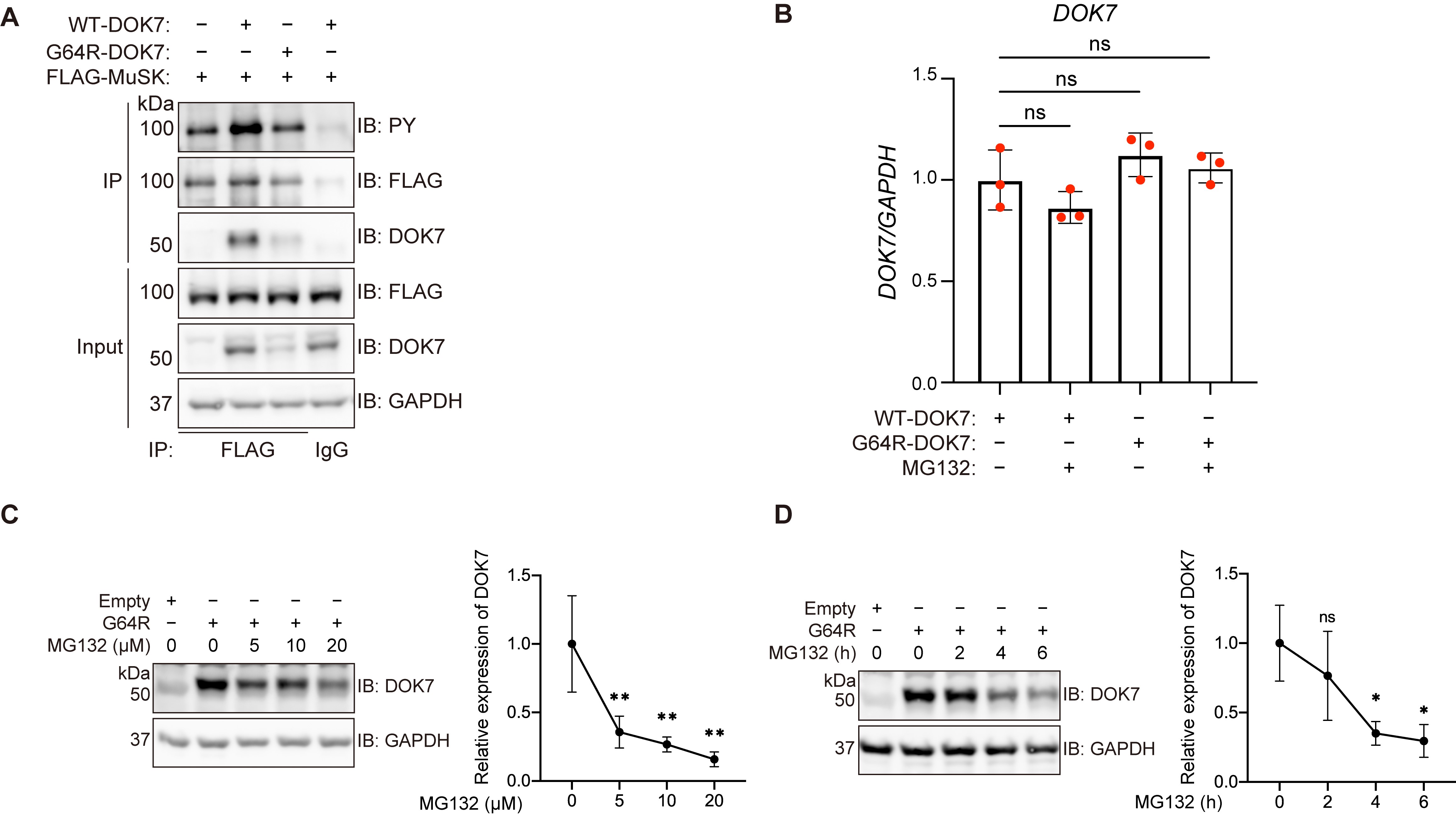

Supplement: Sup_Figure_1_ddac306 [file sup_figure_1_ddac306.jpeg]

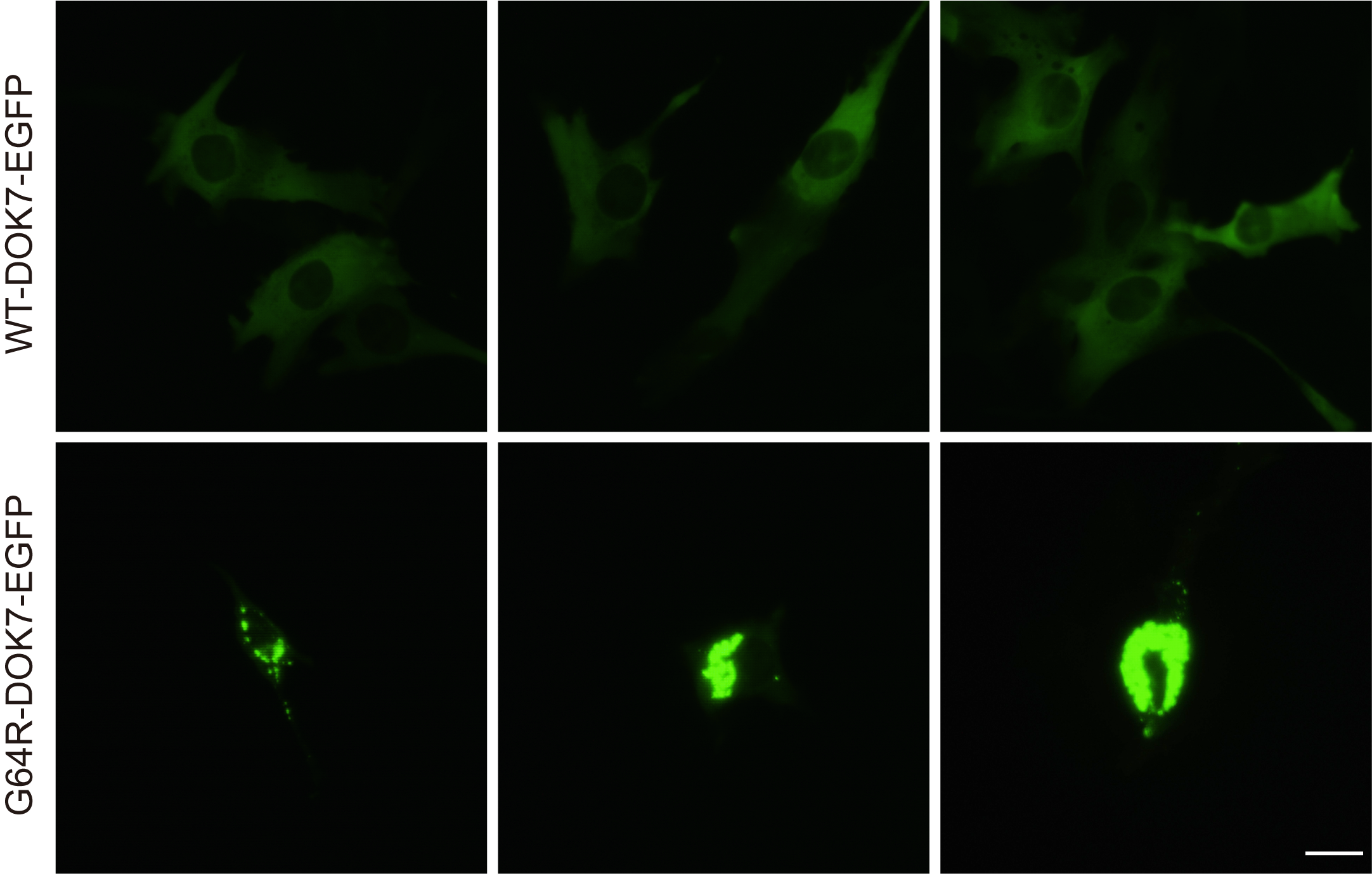

Supplement: Sup_Figure_2_ddac306 [file sup_figure_2_ddac306.zip › Sup_Figure_2_ddac306.png]

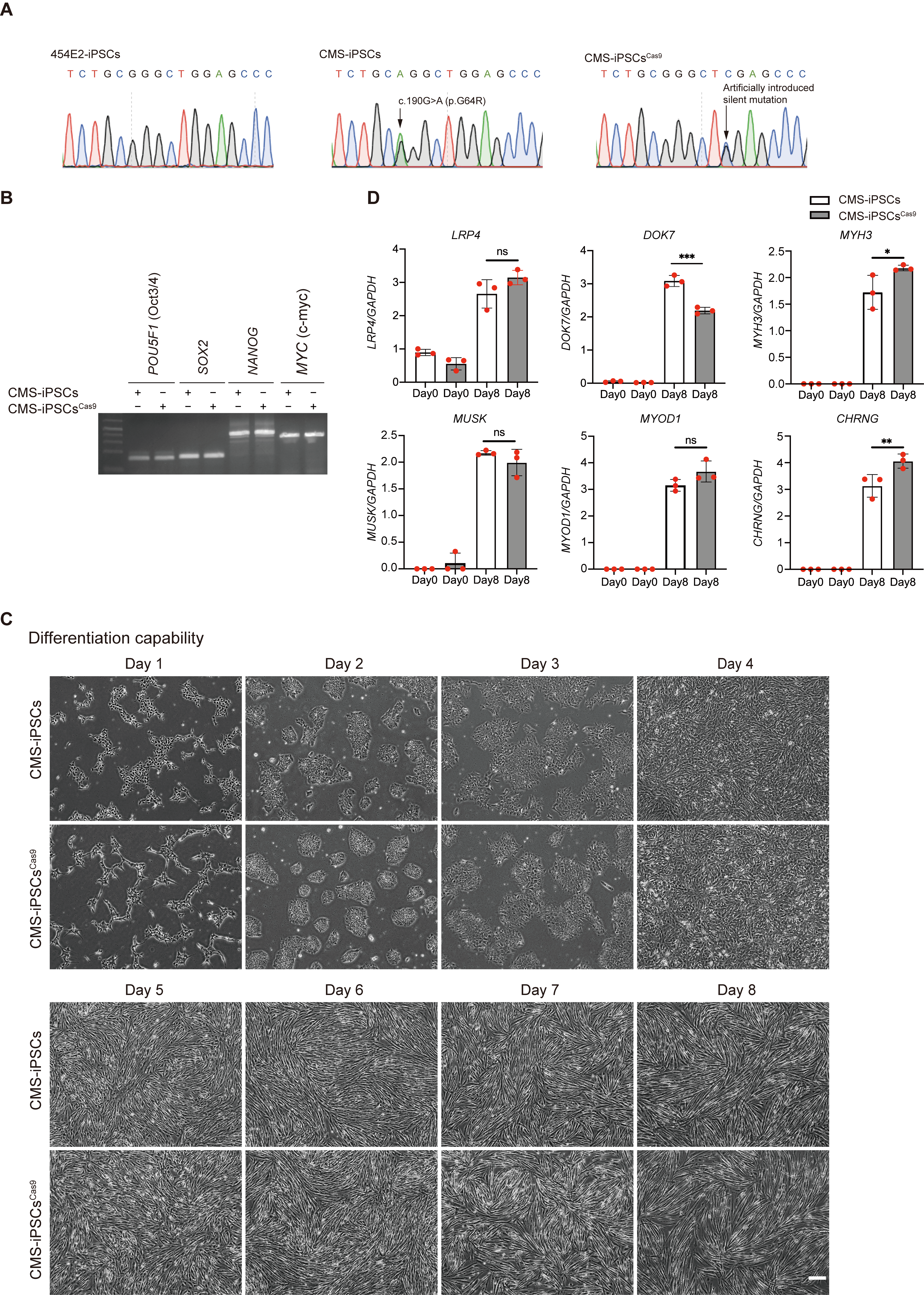

Supplement: Sup_Figure_3_ddac306 [file sup_figure_3_ddac306.zip › Sup_Figure_3_ddac306.png]

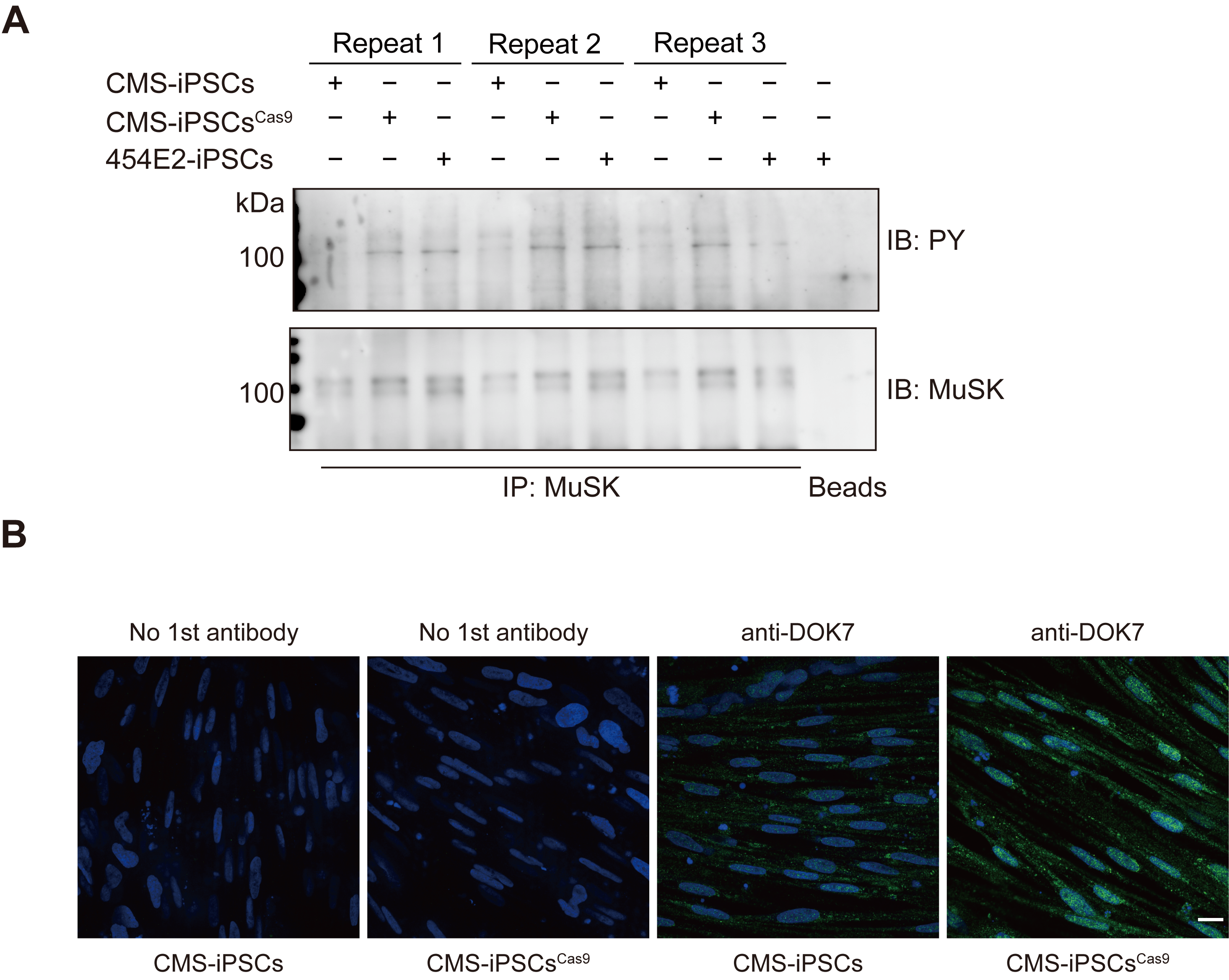

Supplement: Sup_Figure_4_ddac306 [file sup_figure_4_ddac306.zip › Sup_Figure_4_ddac306.png]

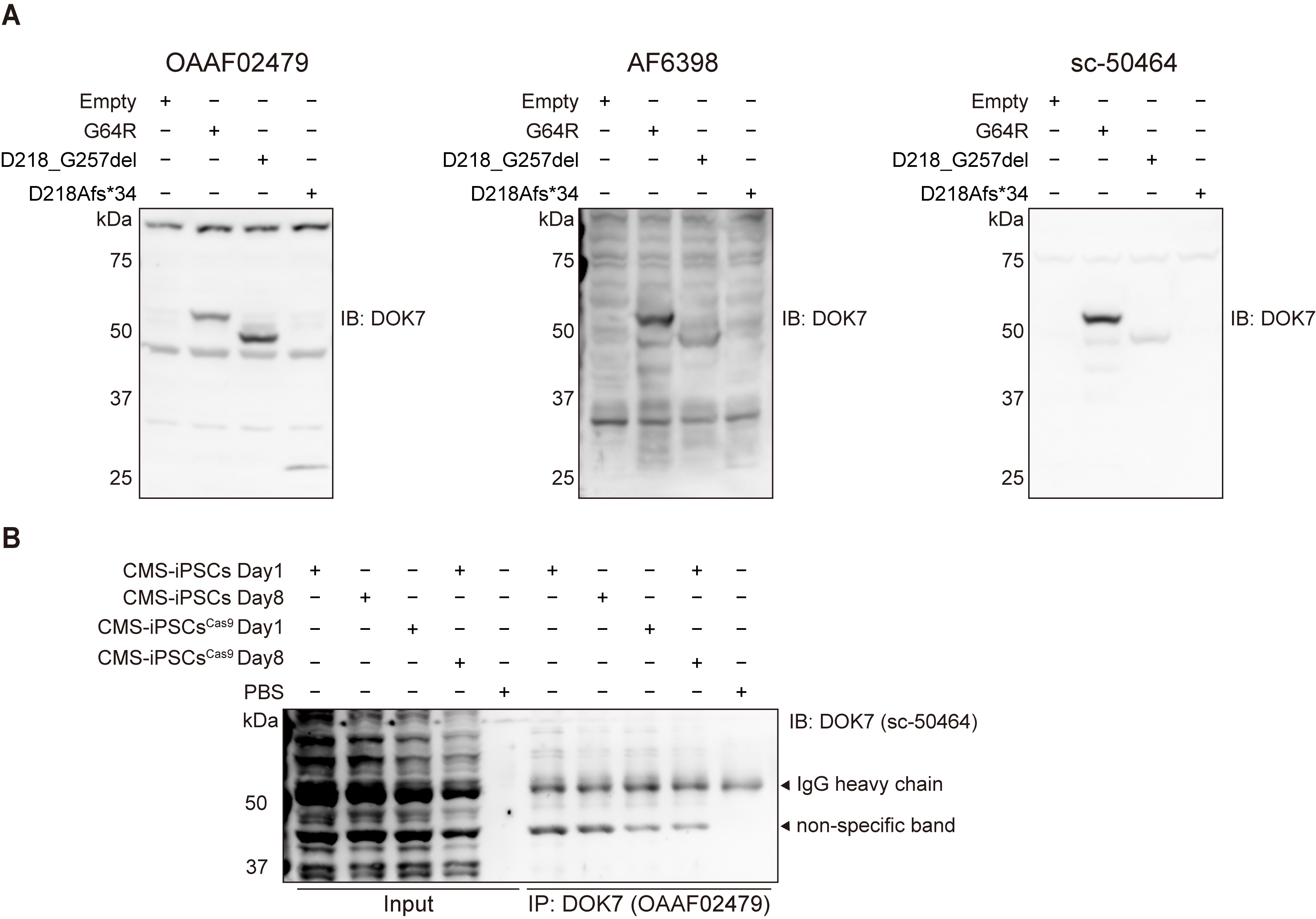

Supplement: Sup_Figure_5_ddac306 [file sup_figure_5_ddac306.jpeg]

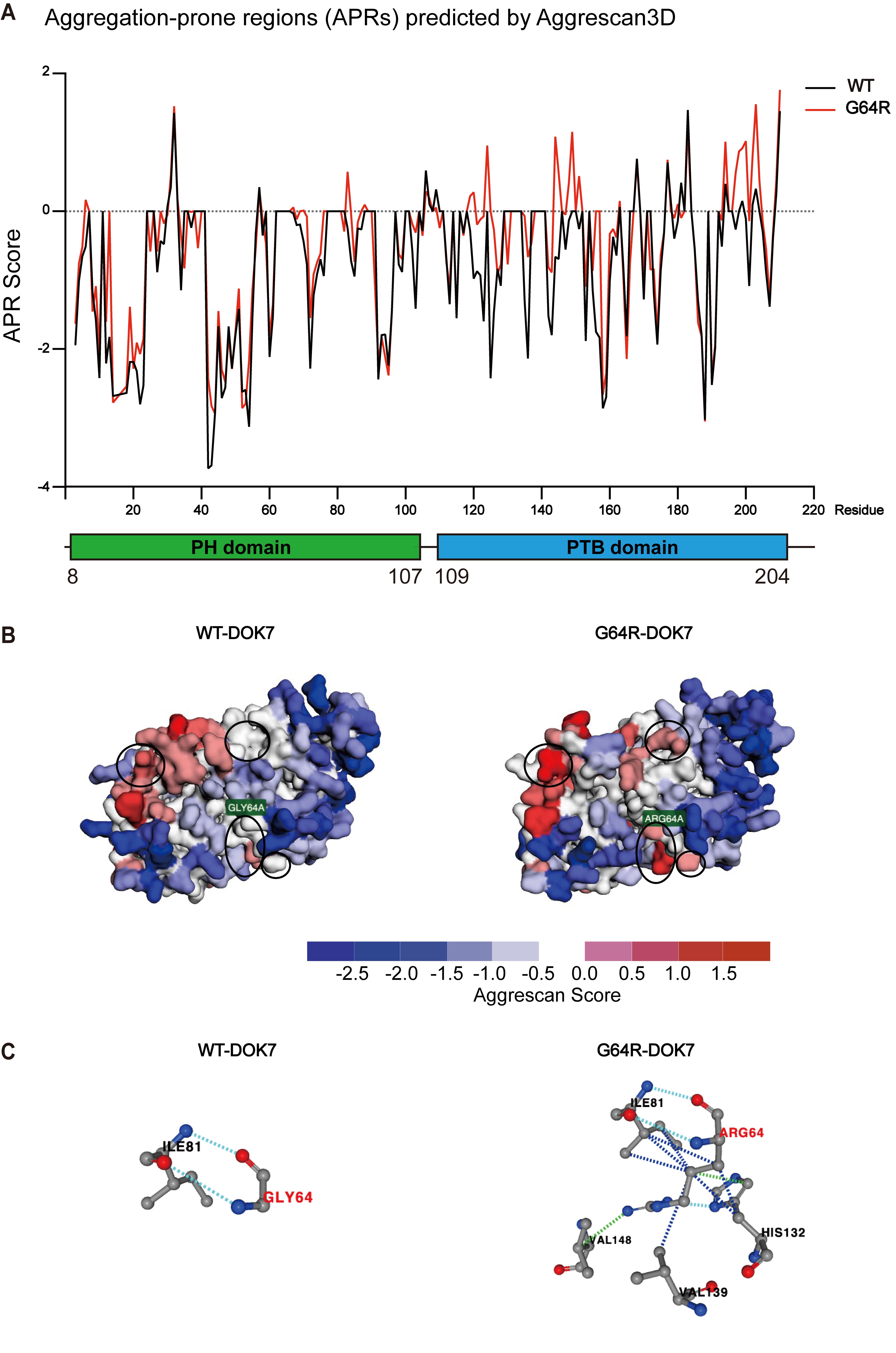

Supplement: Sup_Figure_6_ddac306 [file sup_figure_6_ddac306.jpeg]

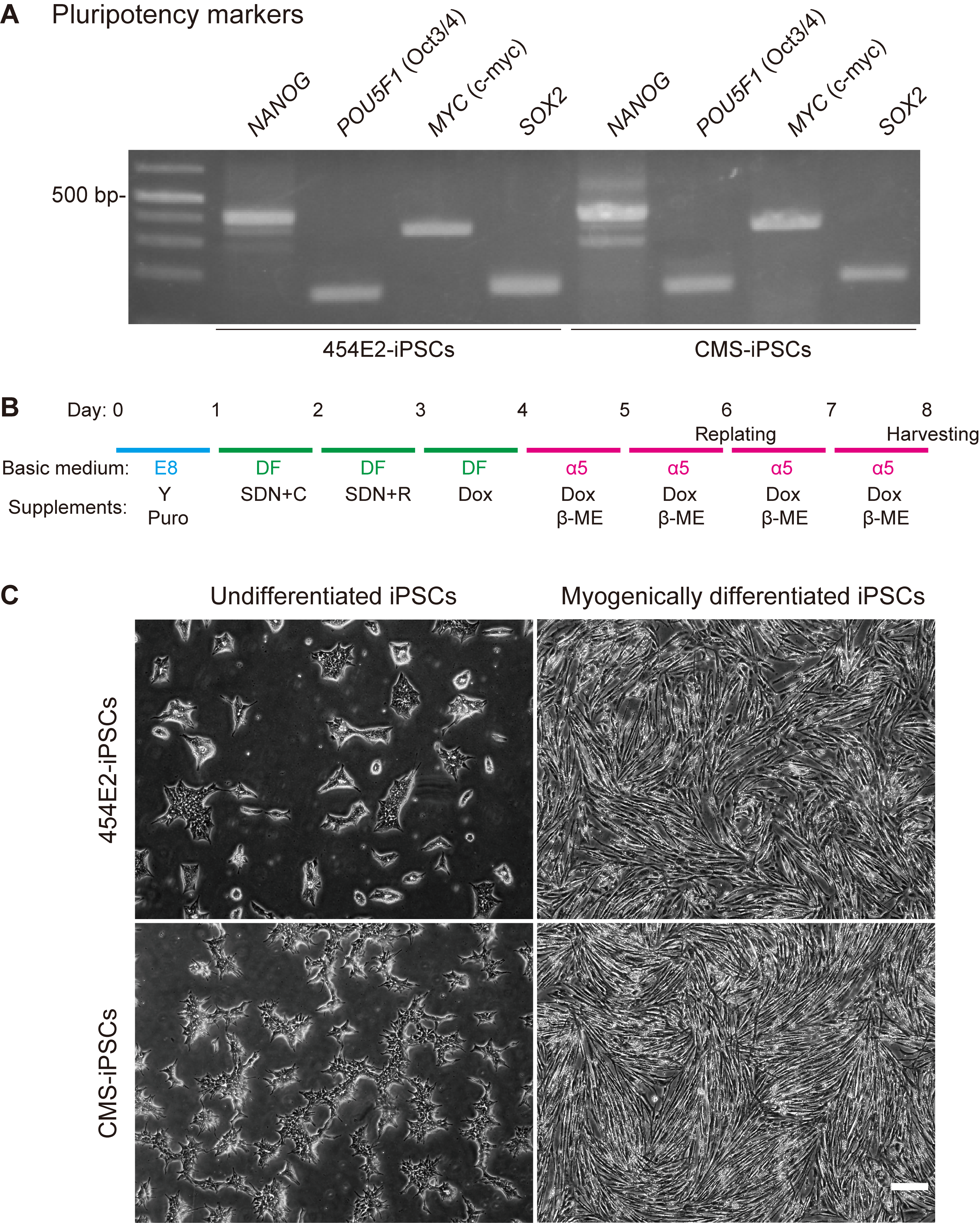

Supplement: Sup_Figure_7_ddac306 [file sup_figure_7_ddac306.zip › Sup_Figure_7_ddac306.png]

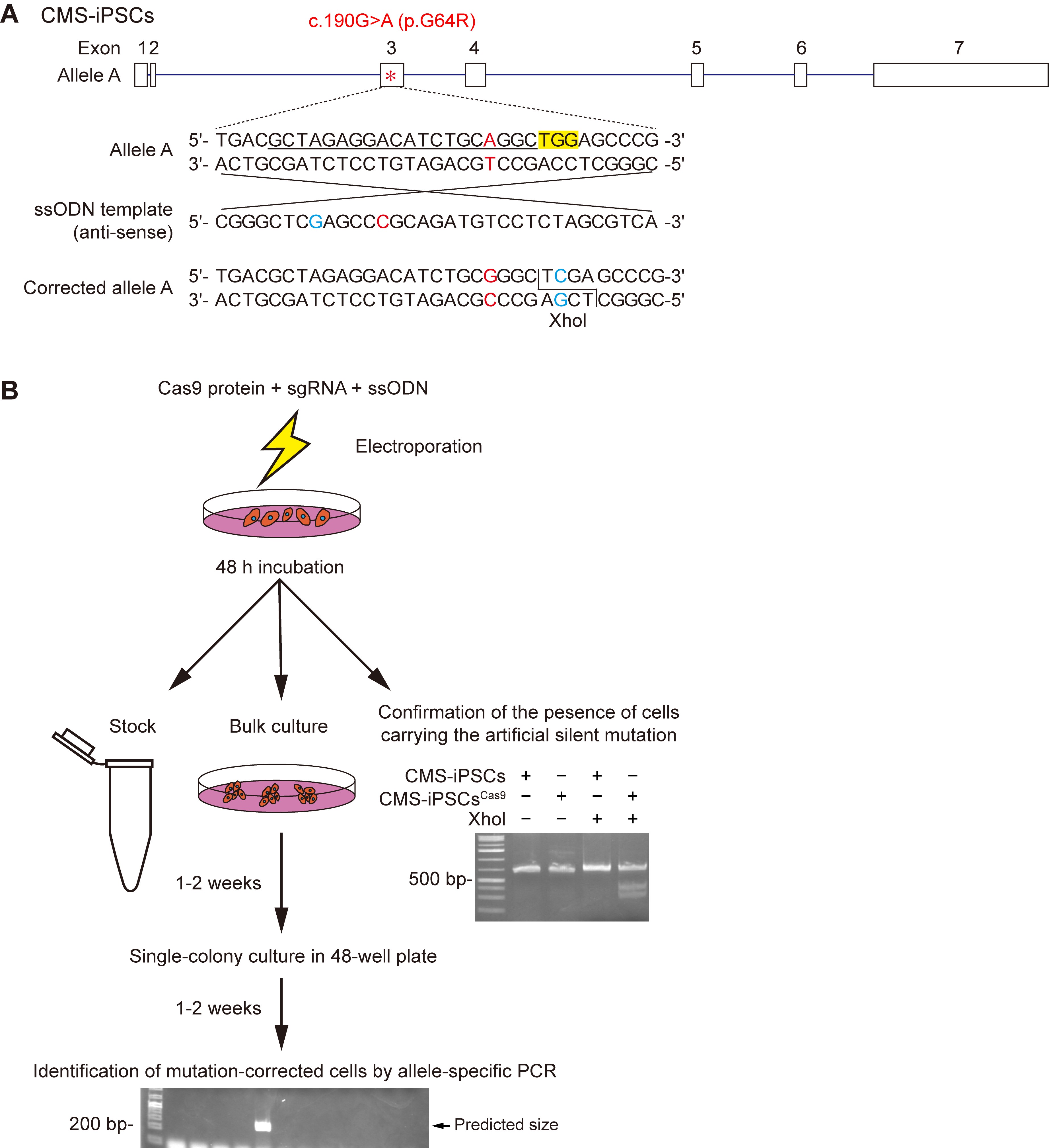

Supplement: Sup_Figure_8_ddac306 [file sup_figure_8_ddac306.jpeg]
