# Supplementary material for: A mutation in DOK7 in congenital myasthenic syndrome forms aggresome in cultured cells, and reduces DOK7 expression and MuSK phosphorylation in patient-derived iPS cells
Source: Hum Mol Genet. 2022 Dec 29;32(9):1511–23. doi: 10.1093/hmg/ddac306 (PMC10117378; doi:10.1093/hmg/ddac306)
Supplement: Sup_Figure_9_ddac306 [file sup_figure_9_ddac306.docx]

1 macro

2 run("Clear Results");

3 run("Set Scale...", "distance=312 known=100 unit=um");

4 run("8-bit");

5 setAutoThreshold ("Huang");

6 //run("Threshold...");

7 setThreshold (25, 255);

8 //setThreshold (25, 255);

9 setOption ("BlackBackground",false);

10 run("Convert to Mask");

11 run("Analyze Particles...", "size=4 show=Outlines display summarize");

12 close();

13 close();
