# Supplementary material for: A mutation in DOK7 in congenital myasthenic syndrome forms aggresome in cultured cells, and reduces DOK7 expression and MuSK phosphorylation in patient-derived iPS cells
Source: Hum Mol Genet. 2022 Dec 29;32(9):1511–23. doi: 10.1093/hmg/ddac306 (PMC10117378; doi:10.1093/hmg/ddac306)
Supplement: Supplementary_Table_S2_ddac306 [file supplementary_table_s2_ddac306.docx]

**Supplementary Table S2. Three commercially available antibodies for DOK7.**

| **Antibody** | **Supplier** | **Host** | **Clonality** | **Epitope (Amino acid positions)** | **Applications in data sheet** | **Applications in our study** |
| --- | --- | --- | --- | --- | --- | --- |
| OAAF02479 | Aviva Systems Biology | Rabbit | Polyclonal | 10-59 | WB, IHC, IF, ELISA | IF, WB |
| AF6398 | R&D Systems | Goat | Polyclonal | 179-299 | WB | IP |
| sc-50464 | Santa Cruz Biotechnology | Rabbit | Polyclonal | 210-287 | WB, IP, IF, ELISA | WB |

c.653-1G>C generates two DOK7 molecules: exon 6-skipped DOK7 and truncated DOK7 (Fig. 2D). OAAF02479 recognizes both exon 6-skipped and truncated DOK7. In contrast, AF6398 and sc-50464 weakly recognize both exon 6-skipped and truncated DOK7 (Supplementary Fig. S7A). AF6398 was previously used for immunoprecipitation (IP) and Western blotting (WB) of human, mouse, and rat DOK7 (1, 2). sc-50464 was previously used for WB of human DOK7 (3, 4). IF, immunofluorescece; IHC, immunohistochemistry; and ELISA, enzyme-linked immuno sorbent assay.

**References**

1. Zhao, K., Shen, C., Li, L., Wu, H., Xing, G., Dong, Z., Jing, H., Chen, W., Zhang, H., Tan, Z. *et al.* (2018) Sarcoglycan Alpha Mitigates Neuromuscular Junction Decline in Aged Mice by Stabilizing LRP4. *J Neurosci*, **38**, 8860-8873.

2. Oury, J., Zhang, W., Leloup, N., Koide, A., Corrado, A.D., Ketavarapu, G., Hattori, T., Koide, S. and Burden, S.J. (2021) Mechanism of disease and therapeutic rescue of Dok7 congenital myasthenia. *Nature*, **595**, 404-408.

3. Beeson, D., Higuchi, O., Palace, J., Cossins, J., Spearman, H., Maxwell, S., Newsom-Davis, J., Burke, G., Fawcett, P., Motomura, M. *et al.* (2006) Dok-7 mutations underlie a neuromuscular junction synaptopathy. *Science*, **313**, 1975-1978.

4. Okada, K., Inoue, A., Okada, M., Murata, Y., Kakuta, S., Jigami, T., Kubo, S., Shiraishi, H., Eguchi, K., Motomura, M. *et al.* (2006) The muscle protein Dok-7 is essential for neuromuscular synaptogenesis. *Science*, **312**, 1802-1805.
