# Supplementary material for: A mutation in DOK7 in congenital myasthenic syndrome forms aggresome in cultured cells, and reduces DOK7 expression and MuSK phosphorylation in patient-derived iPS cells
Source: Hum Mol Genet. 2022 Dec 29;32(9):1511–23. doi: 10.1093/hmg/ddac306 (PMC10117378; doi:10.1093/hmg/ddac306)
Supplement: Supplementary_Table_S3_ddac306 [file supplementary_table_s3_ddac306.docx]

**Supplementary Table S3a. *In silico* tools to predict the effects of missense mutations**

| **Name of the tool** | **URL** | **Input** |
| --- | --- | --- |
| Aggrescan3D 2.0 (ΔΔG) (1) | http://biocomp.chem.uw.edu.pl/A3D2/ | PDB ID |
| PremPS (ΔΔG) (2) | https://lilab.jysw.suda.edu.cn/research/PremPS/ | PDB ID |
| DynaMut (ΔΔG) (3) | http://biosig.unimelb.edu.au/dynamut/ | PDB ID |
| MAESTROweb (ΔΔG) (4) | https://pbwww.services.came.sbg.ac.at/maestro/web/maestro/workflow | PDB ID |
| DUET(ΔΔG) (5) | http://biosig.unimelb.edu.au/duet/ | PDB ID |
| PoPMuSiC (ΔΔG) (6) | https://soft.dezyme.com/query/create/pop | PDB ID |
| SAAFEC (ΔΔG) (7) | http://compbio.clemson.edu/SAAFEC-SEQ/ | PDB ID |
| Missense3D (8) | http://missense3d.bc.ic.ac.uk/~missense3d/ | PDB ID |

**Supplementary Table S3b. Calculated ΔΔG by five mutations at the PH domain of DOK7 (kcal/mol)**

| **Mutation** | **Aggrescan3D 2.0** | **PremPS** | **DynaMut** | **MAESTROweb** | **DUET** | **PoPMuSiC** | **SAAFEC** |
| --- | --- | --- | --- | --- | --- | --- | --- |
| P31T | 1.466 | 0.29 | -0.864 | 0.259 | 1.443 | 0.80 | 0.89 |
| A33V | 0.428 | 0.03 | -1.062 | 0.283 | 0.206 | 0.64 | 0.70 |
| G64R | 24.482 | 0.53 | -0.790 | 0.341 | 1.457 | 2.55 | 0.49 |
| Y71F | -0.707 | 0.57 | -0.926 | 0.871 | 0.659 | 0.73 | 0.98 |
| T77M | 2.708 | 0.17 | -2.859 | -0.236 | 0.191 | 1.02 | 0.12 |

ΔΔG > 0 and ΔΔG < 0 represent destabilizing and stabilizing mutations, respectively.

**Supplementary Table S3c. Predicted structural changes by five mutations at the PH domain of DOK7 by Missense3D**

| **Mutation** | **Predicted structural changes** |
| --- | --- |
| P31T | No structural damage detected |
| A33V | No structural damage detected |
| G64R | Clash, Buried charge introduced, Disallowed phi/psi, Buried Gly replaced |
| Y71F | No structural damage detected |
| T77M | Buried H-bond breakage |

**References**

1 Kuriata, A., Iglesias, V., Pujols, J., Kurcinski, M., Kmiecik, S. and Ventura, S. (2019) Aggrescan3D (A3D) 2.0: prediction and engineering of protein solubility. *Nucleic Acids Res*, **47**, W300-W307.

2 Chen, Y., Lu, H., Zhang, N., Zhu, Z., Wang, S. and Li, M. (2020) PremPS: Predicting the impact of missense mutations on protein stability. *PLoS computational biology*, **16**, e1008543.

3 Rodrigues, C.H., Pires, D.E. and Ascher, D.B. (2018) DynaMut: predicting the impact of mutations on protein conformation, flexibility and stability. *Nucleic Acids Res*, **46**, W350-W355.

4 Laimer, J., Hiebl-Flach, J., Lengauer, D. and Lackner, P. (2016) MAESTROweb: a web server for structure-based protein stability prediction. *Bioinformatics*, **32**, 1414-1416.

5 Pires, D.E., Ascher, D.B. and Blundell, T.L. (2014) DUET: a server for predicting effects of mutations on protein stability using an integrated computational approach. *Nucleic Acids Res*, **42**, W314-319.

6 Pucci, F., Bernaerts, K.V., Kwasigroch, J.M. and Rooman, M. (2018) Quantification of biases in predictions of protein stability changes upon mutations. *Bioinformatics*, **34**, 3659-3665.

7 Getov, I., Petukh, M. and Alexov, E. (2016) SAAFEC: Predicting the Effect of Single Point Mutations on Protein Folding Free Energy Using a Knowledge-Modified MM/PBSA Approach. *Int J Mol Sci*, **17**, 512.

8 Ittisoponpisan, S., Islam, S.A., Khanna, T., Alhuzimi, E., David, A. and Sternberg, M.J.E. (2019) Can Predicted Protein 3D Structures Provide Reliable Insights into whether Missense Variants Are Disease Associated? *J Mol Biol*, **431**, 2197-2212.
