# Supplementary material for: A mutation in DOK7 in congenital myasthenic syndrome forms aggresome in cultured cells, and reduces DOK7 expression and MuSK phosphorylation in patient-derived iPS cells
Source: Hum Mol Genet. 2022 Dec 29;32(9):1511–23. doi: 10.1093/hmg/ddac306 (PMC10117378; doi:10.1093/hmg/ddac306)
Supplement: Supplementary_Table_S4_ddac306 [file supplementary_table_s4_ddac306.docx]

**Supplementary Table S4. Primers**

|  | **Primers** | **Sequence (5′-3′)** | **Size** |
| --- | --- | --- | --- |
| **Primers for quantitative RT-PCR** | |  |  |
|  | Downstream of tyrosine kinase 7 (*DOK7*) | GCCATCATGCTGGGCTTTGACA | 116 bp |
|  |  | AACTTGGTGCCTGGAGCCACTG |  |
|  | Low-density lipoprotein receptor-related protein 4 (*LRP4*) | GTGTGGCAGAACCTTGACAGTC | 100 bp |
|  |  | ACCGCTCTAACTTGGCATTCTCC |  |
|  | Muscle-specific kinase (*MUSK*) | TTTGCTGTCCGTGCCAGAATGC | 132 bp |
|  |  | GGCTTTGAGGACGTCATTGGTG |  |
|  | Myogenic differentiation 1 (*MYOD1*) | CACTCCGGTCCCAAATGTAG | 180 bp |
|  |  | TTCCCTGTAGCACCACACAC |  |
|  | Myosin heavy chain 3 (*MYH3*) | GCAGATTGAGCTGGAAAAGG | 167 bp |
|  |  | TCAGCTGCTCGATCTCTTCA |  |
|  | Cholinergic receptor nicotinic gamma subunit (*CHRNG*) | CACCAACCTCATCTCCCTGA | 202 bp |
|  |  | GAGGGCCACCTCGAAGACAC |  |
|  | Glyceraldehyde 3-phosphate dehydrogenase (*GAPDH*) | GTCTCCTCTGACTTCAACAGCG | 131 bp |
|  |  | ACCACCCTGTTGCTGTAGCCAA |  |
| **Primers for RT-PCR of pluripotency markers** | |  |  |
|  | c-myc myelocytomatosis viral oncogene homolog (*MYC*) | GCGTCCTGGGAAGGGAGATCCGGAGC | 328 bp |
|  |  | TTGAGGGGCATCGTCGCGGGAGGCTG |  |
|  | Octamer-binding transcription factor 3/4 (*POU5F1*) | GACAGGGGGAGGGGAGGAGCTAGG | 144 bp |
|  |  | CTTCCCTCCAACCAGTTGCCCCAAAC |  |
|  | Nanog homeobox (*NANOG*) | CAGCCCCGATTCTTCCACCAGTCCC | 391 bp |
|  |  | CGGAAGATTCCCAGTCGGGTTCACC |  |
|  | Sex determining region Y-box 2 (*SOX2*) | GGGAAATGGGAGGGGTGCAAAAGAGG | 151 bp |
|  |  | TTGCGTGAGTGTGGATGGGATTGGTG |  |
| **Primers for analysis of *DOK7*** | |  |  |
|  | *DOK7* exon 3 | GAGTTCATTTCCGCTCTTGC | 700 bp |
|  |  | TGGGTTAAAGGCTCCAGTTG |  |
|  | *DOK7* exon 6 | GCCTTGGGCACAGTATGAGT | 685 bp |
|  |  | CTCTCCTGCTGGAGGCTTAG |  |
|  | *DOK7* exon 4/5 (spanning exon junction)-7 | GTGGGTACTGGGCTGGCGTCTTCT | 537 bp |
|  |  | ACGAGAGGCTGCTGGAGTAA |  |
|  | *DOK7* Cas9 (allele-specific primers for Cas9-corrected colony) | GCCTGACGCTAGAGGACATCTGCGGGGTC | 246 bp |
|  |  | ACCTCTGCAGCCCACGTTGGTGAGGGGC |  |
