# Supplementary material for: A mutation in DOK7 in congenital myasthenic syndrome forms aggresome in cultured cells, and reduces DOK7 expression and MuSK phosphorylation in patient-derived iPS cells
Source: Hum Mol Genet. 2022 Dec 29;32(9):1511–23. doi: 10.1093/hmg/ddac306 (PMC10117378; doi:10.1093/hmg/ddac306)
Supplement: Legends_for_Supplementary_Figures_ddac306 [file legends_for_supplementary_figures_ddac306.docx]

**Legends for Supplementary Figures**

**Supplementary Figure S1.** **Effects of p.G64R-DOK7 on MuSK phosphorylation, mRNA expression, and protein solubility after MG132 treatment. (A)** FLAG-MuSK were co-transfected with wild-type (WT)-DOK7 or p.G64R-DOK7 into COS7 cells. At 24 h after transfection, cell lysates were immunoprecipitated (IP) by anti-FLAG antibody, and followed by immunoblotting (IB) with the indicated antibody. PY, anti-phosphotyrosine antibody. (**B**) At 18 h after transfection of COS7 cells with WT-DOK7 or p.G64R-DOK7, 10 μM MG132 was added for 3 h. Red dots indicate the ratio of *DOK7* mRNA normalized for *GAPDH* mRNA (*n* = 3 independent experiments). *P*-value was 0.08 by one-way ANOVA, and Dunnett’s multiple comparison test yielded no significance (ns) compared to WT-DOK7. (**C and D**) Representative Western blotting and quantitative analysis of DOK7 and GAPDH in soluble fractions in transfected COS7 cells after MG132 treatment. (**C**) Indicated concentrations of MG132 were added 3 h before harvesting cells. **(D)** Cells were harvested at the indicated time points after adding 20 µM MG132. Expression levels were normalized to that of GAPDH, and also to the ratio of wild-type (WT)-DOK7 at 0 µM MG132 **(C)**, or to the ratio at time 0 after adding MG132 **(D)**. Mean and SD (*n* = 3 experiments) are indicated with individual values in red dots. One-way ANOVA with Dunnett's post hoc multiple comparison test was applied to compare to a value at 0 µM MG132 **(C)** and at time 0 after adding MG132 **(D)** (ns, no significance, **p* < 0.05, ***p* < 0.01).

**Supplementary Figure S2. Effects of p.G64R on protein aggregation in transfected C2C12 cells.** Representative immunofluorescent images of WT-DOK7-EGFP and p.G64R-DOK7-EGFP in transfected C2C12 cells. Scale bar = 20 μm.

**Supplementary Figure S3. Myogenic differentiation of iPSCs.** (**A**) RT-PCR of pluripotency markers in control 454E2-iPSCs and patient-derived CMS-iPSCs. (**B**) Schematic illustration showing the workflow of iPSCs differentiation into myogenic cells. E8: Essential 8 Medium; DF: DMEM/F12 with 5% KSR; α5: αMEM with 5% KSR; Puro: puromycin; Y: Y27632; SDN: SB431542, dorsomorphin, and N-acetyl-L-cysteine; C: CHIR99021; R: retinoic acid; Dox: doxycycline; and β-ME: β-mercaptoethanol. (**C**) Representative phase-contrast images of undifferentiated and myogenically differentiated iPSCs on day 8. Scale bar = 200 μm.

**Supplementary Figure S4. Representative immunostaining of DOK7 and MuSK phosphorylation** in CMS-iPSCs and CMS-iPSCs^Cas9^**.** (**A**) DOK7 (green) and with DAPI (blue) were immunostained in myogenically differentiated isogenic CMS-iPSCs^Cas9^ and patient-derived CMS-iPSCs on day 8. Scale bar = 20 μm. (**B**) Three additional sets of Western blotting showing MuSK phosphorylation in CMS-iPSCs and CMS-iPSCs^Cas9^, the condition of which is identical to that shown in Figure 6C. The experiments were performed in quadruplicate in all, because the low amount of phosphorylated MuSK hindered us from performing quantitative analysis.

**Supplementary Figure S5. Western blotting with three commercially available anti-DOK7 antibodies. (A)** Representative Western blotting of DOK7-transfected COS7 cells by three anti-DOK7 antibodies indicated in Supplementary Table S1. Epitopes of these antibodies are schematically shown in Figure 2D. Note that OAAF02479 recognizes three mutant DOK7 proteins, whereas AF6398 and sc-50464 recognize p.G64R-DOK7 but scarcely recognize p.D218_G257del and p.D218Afs*34. (**B**) Representative Western blotting of DOK7 in myogenically differentiated iPSCs on day 8. DOK7 was immunoprecipitated (IP) by OAAF02479 and probed with sc-50464. In Figure 6D, DOK7 was immunoprecipitated by AF6398 and probed with sc-50464. Note that, in contrast to Figure 6D, DOK7 was not detected in any cells after immunoprecipitation in this panel.

**Supplementary Figure S6. *In silico* analysis by Aggrescan3D and PremPS**. (**A**) Plot of Aggrescan3D scores. (**B**) Structures of mouse wild-type (WT)-DOK7 in the protein database (PDB: 3ML4) and p.G64R-DOK7 predicted by Aggrescan3D. Circles show amino acids that showed increased aggregation propensity by p.G64R. (**C**) Non-covalent interactions of WT-DOK7 (PDB: 3ML4) and p.G64R DOK7 predicted by PremPS. Van der Waals force, hydrophobic interaction, and hydrogen bond are indicated by dotted lines in green, dark blue, and light blue, respectively.

**Supplementary Figure S7.** **Establishment of the isogenic iPSCs.** (**A**) Sanger sequencing of *DOK7* exon 3 of 454E2 (control)-iPSCs, CMS-iPSCs, and CMS-iPSCs^Cas9^. (**B**) RT-PCR of pluripotency markers in CMS-iPSCs and CMS-iPSCs^Cas9^. (**C**) Representative phase-contrast microscopic images of myogenically differentiated CMS-iPSCs and CMS-iPSCs^cas9^ for 8 days. **(D)** Quantitative RT-PCR of myogenic markers (*MYH3* and *MYOD1*) and NMJ-associated genes (*LRP4*, *MUSK*, *DOK7*, and *CHRNG*) in myocyte-differentiated iPSCs on day 8. Mean and SD (*n* = 3 independent experiments) are indicated. One-way ANOVA and Dunnett's multiple comparison test (ns, no significance; **p* < 0.05, ***p* < 0.01, and ****p*< 0.001).

**Supplementary Figure S8. Generation of isogenic iPS cell line by CRISPR/Cas9.** (**A**) Schematic representation of the target of CRISPR/Cas9 on an allele carrying c.190G>A (allele A) in *DOK7* exon 3 in CMS iPSCs. We attempted to introduce wild-type nucleotide at c.190G>A (red) and an artificial silent mutation (blue), which ablates the 3-bp protospacer-adjacent motif (PAM) site (highlighted) and generates a restriction site (CTCGAG) of Xhol. The length of the antisense ssODN was 81 nucleotides, and only the target sequence is indicated. The target site for guide RNA is underlined. (**B**) Flowchart showing the establishment of an isogenic cell line, CMS-iPSCs^cas9^. The presence of cells carrying the artificial silent mutation was confirmed by restriction digestion with XhoI at 48 h after electroporation. A colony comprised of CMS-iPSCs^cas9^ was identified by allele-specific PCR.

**Supplementary Figure S9. ImageJ macro for the analysis of AChR clusters in C2C12 myotubes.**
