# Supplementary material for: A mutation in DOK7 in congenital myasthenic syndrome forms aggresome in cultured cells, and reduces DOK7 expression and MuSK phosphorylation in patient-derived iPS cells
Source: Hum Mol Genet. 2022 Dec 29;32(9):1511–23. doi: 10.1093/hmg/ddac306 (PMC10117378; doi:10.1093/hmg/ddac306)
Supplement: Supplementary_Materials_and_Methods_ddac306 [file supplementary_materials_and_methods_ddac306.docx]

**Supplementary Materials and Methods**

**Genomic sequencing of the patient**

The study was approved by the ethical review committees of the National Center for Neurology and Psychiatry (A2017-061) and the Nagoya University (2007-0598-3 and 2014-0136-5), and was performed in accordance with the relevant guidelines. Signed informed consents were obtained from the patient and the parents. Sanger sequencing was performed on the *CHRNA1, CHRNB1, CNRND, CHRNE, COLQ, RAPSN, and DOK7* genes*.*

**Expression analysis of wild-type and mutant DOK7 in COS7 cells**

COS7 cells were maintained in DMEM supplemented with 10% FBS at 37°C with 5% CO_2_. One day before transfection, cells were seeded at a density of 2.0 ×10^5^ cells per well in 6-well plate. On the following day, cells were transfected with 1 μg of plasmid encoding either WT-DOK7 or p.G64R-DOK7 with or without 1 μg of plasmid encoding FLAG-MuSK. Transfection was performed with FuGENE 6 (Promega) according to the manufacturer’s instructions. For immunofluorescence staining, 1.0 × 10^5^ cells were plated on a collagen I–coated glass coverslip in each well of a 6-well plate before transfection.

**Coimmunoprecipitation**

For the coimmunoprecipitation assay, whole-cell lysates were prepared as stated above. The cell lysate containing 200 μg protein was incubated with 1 μg anti-FLAG M2 antibody (F3290, Sigma-Aldrich) or anti-DOK7 antibody (AF6398, R&D Systems) at 4℃ for 1 h with gentle rotation. Dynabeads Protein G (Thermo Fisher Scientific) was washed three times with 500 µl PBS containing protease inhibitors and phosphatase inhibitors as described above. Then, 30 μl of the beads were added to the lysate mixture and incubated at 4℃ overnight with gentle rotation. The beads were washed three times with 500 μl PLC buffer containing protease inhibitors and phosphatase inhibitors. Then, the beads were resuspended in 30 μl Laemmli buffer, and heated at 95°C for 5 min. Finally, Dynabeads were removed by the Standard DYNAL Invitrogen Bead Separator Magnetic Rack (Thermo Fisher Scientific), and the samples were subjected to Western blotting.

**Plasmid constructions**

The full-length human *DOK7* encoding transcript variant 1 (NM_173660.5) was cloned into pcDNA3.1(+). Seven mutations [c.91C>A (p.P31T), c.98C>T (p.A33V), c.190G>A (p.G64R), c.212A>T (p.Y71F), c.230C>T (p.T77M), c.653_772del (p.D218_G257del) due to skipping of exon 6, and c.653_659delACCCAAG (p.D218Afs*34) due to an activation of a cryptic 3’ splice site] were introduced into pcDNA3.1-DOK7 using the QuikChange Site-Directed Mutagenesis kit (Agilent). Presence of the mutation and absence of artifacts were confirmed by sequencing the entire inserts.

A plasmid encoding EGFP-fused DOK7 was generated as previously described (49). Briefly, *DOK7* cDNA was cloned into the pEGFP-N1 (Clontech #6085-1) plasmid at the HindIII and KpnI sites. A 12-amino-acid linker (TVPRARDPPVAT) was inserted between *DOK7* cDNA and *EGFP* cDNA to allow proper folding. c.190G>A (p.G64R) was introduced into the plasmid using the QuikChange Site-Directed Mutagenesis kit. The integrity of plasmid was confirmed by sequencing the entire insert.

Human *MUSK* cDNA was cloned into p3xFLAG-CMV-14 plasmid at the EcoRI and XbaI sites, and was used for coimmunoprecipitation assay.

**mRNA expression analysis by reverse-transcription PCR (RT-PCR) and real-time RT-PCR**

RNA was isolated from cultured cells with RNeasy Mini Kit (Qiagen) according to the manufacturer’s instructions. RNA was reverse-transcribed using random hexamers (Thermo Fisher Scientific) and ReverTraAce (Toyobo). For RT-PCR, PCR amplification was carried out using GoTaq (Promega) with 30 cycles. RT-PCR products were excised from an agarose gel, and were purified with Wizard SV gel and PCR clean-up system (Promega). The purified RT-PCR products were cloned into pGEM-T Easy Vector (Promega), and were subjected to Sanger sequencing. Real-time RT-PCR was performed with the LightCycler 480 (Roche Diagnostics) using the TB Green Premix ExTaq II (Takara Bio). Primer sequences used for RT-PCR and real-time RT-PCR are shown in Supplementary Table S1.

**Western blotting**

Whole-cell lysates were prepared with 300 μl PLC buffer [50 mM HEPES pH 7.0, 150 mM NaCl, 10% (vol/vol) glycerol, 1% (vol/vol) TritonX-100, 1.5 mM MgCl_2_, 1 mM EGTA, 100 mM NaF, 10 mM sodium pyrophosphate] supplemented with protease inhibitors (1 μg/μl pepstatin A, 1 μg/μl Aprotinin, 1 μg/μl Leupeptin, 1 mM PMSF) and phosphatase inhibitors (PhosSTOP, Roche Life Science). Then, the cell lysates were gently rotated at 4℃ for 30 min, and centrifuged for 5 min at 15,000 × *g* at 4°C. The Pierce 660 nm Protein Assay was used to measure total protein concentration. Cell lysates were heated at 95°C for 5 min in 2x Laemmli buffer, resolved by a 10% sodium dodecyl sulfate-polyacrylamide gel electrophoresis (SDS-PAGE), and transferred to a polyvinylidene fluoride membrane (Immobilon-P, Millipore). After blocking with Tris-buffered saline and 0.05% Tween 20 (TBS-T) containing 3% bovine serum albumin (BSA), the membrane was incubated with a primary antibody at 4°C overnight. The primary antibody was against anti–DOK7 (1:500, sc-50464, Santa Cruz Biotechnology), anti–DOK7 (1:500, OAAF02479, Aviva Systems Biology), anti–GAPDH (1:1000, G9545, Sigma-Aldrich), anti-FLAG M2 (1:500, F3290, Sigma-Aldrich), or anti-phosphotyrosine (1:1000, 4G10, Upstate) antibody. The membranes were rinsed with TBS-T and incubated with horseradish peroxidase (HRP)-conjugated goat anti-rabbit IgG (1:2000, 7074, Cell Signaling Technology) or horse anti-mouse IgG (1:2000, 7076, Cell Signaling Technology) for 1 h at room temperature. Immunoreactive proteins were visualized using ECL (GE Healthcare). The band intensities were quantified with the ImageJ software.

**Immunofluorescence staining and confocal microscope**

The cells were fixed with 4% paraformaldehyde for 10 min at room temperature and permeabilized with 0.1% Triton X-100 in PBS for 10 min at room temperature. Then, cells were blocked with Blocking One (Nacalai Tesque) for 1 h at room temperature and subsequently incubated overnight at 4℃ with a primary antibody. The primary antibody was against DOK7 (1:300, OAAF02479, Aviva Systems Biology), HSP70 (1:200, ADI-SPA-810, Enzo Life Sciences), ubiquitin (1:500, sc-8017, Santa Cruz Biotechnology), parkin (1:200, 4211, Cell Signaling Technology), P62 (1:200, GP62-C, Progene Biotechnik) or α-Tubulin (1:200, 3873, Cell Signaling Technology). On the following day, cells were incubated with a secondary antibody, Alexa Flour 594-tagged anti-mouse IgG or Alexa Flour 594-tagged anti-guinea pig IgG, with or without Alexa Fluor 488-tagged anti-rabbit IgG. Images of the stained cells were acquired using a confocal ultrahigh-resolution microscope (SpinSR10, Olympus). All images were processed with OlyVIA software (Olympus). Five visual fields were randomly taken for each sample. For calculating the ratio of cells containing aggregates in variable combinations of MG132 (ab141003, Abcam) and nocodazole (487928, Sigma), more than 80 cells of each sample were counted.
